# The mutational landscape of chromatin regulatory factors across 4,623 tumor samples

**DOI:** 10.1186/gb-2013-14-9-r106

**Published:** 2013-09-24

**Authors:** Abel Gonzalez-Perez, Alba Jene-Sanz, Nuria Lopez-Bigas

**Affiliations:** 1 Research Unit on Biomedical Informatics, Department of Experimental and Health Sciences, Universitat Pompeu Fabra, Dr. Aiguader 88, Barcelona, Spain; 2 Institució Catalana de Recerca i Estudis Avançats (ICREA), Barcelona, Spain

## Abstract

**Background:**

Chromatin regulatory factors are emerging as important genes in cancer development and are regarded as interesting candidates for novel targets for cancer treatment. However, we lack a comprehensive understanding of the role of this group of genes in different cancer types.

**Results:**

We have analyzed 4,623 tumor samples from thirteen anatomical sites to determine which chromatin regulatory factors are candidate drivers in these different sites. We identify 34 chromatin regulatory factors that are likely drivers in tumors from at least one site, all with relatively low mutational frequency. We also analyze the relative importance of mutations in this group of genes for the development of tumorigenesis in each site, and in different tumor types from the same site.

**Conclusions:**

We find that, although tumors from all thirteen sites show mutations in likely driver chromatin regulatory factors, these are more prevalent in tumors arising from certain tissues. With the exception of hematopoietic, liver and kidney tumors, as a median, the mutated factors are less than one fifth of all mutated drivers across all sites analyzed. We also show that mutations in two of these genes, *MLL* and *EP300*, correlate with broad expression changes across cancer cell lines, thus presenting at least one mechanism through which these mutations could contribute to tumorigenesis in cells of the corresponding tissues.

## Background

Highly conserved molecular mechanisms are responsible for maintaining genome integrity and tightly regulated gene expression, which is essential for cell survival. Those include the fine regulation of chromatin structure, mainly maintained through three distinct processes: the post-translational modification of histone tails, the replacement of core histones by histone variants, and the direct structural remodeling by ATP-dependent chromatin-remodeling enzymes [1]. The proteins that control this system, broadly referred to as chromatin regulatory factors (CRFs), contribute to the establishment of chromatin structures that modulate the expression of large gene sets, either by establishing more inaccessible regions or by placing histone marks that open the chromatin and allow the binding of other factors. These CRFs help to maintain cellular identity, and mutations in them (commonly called epimutations) often lead to a de-regulation of gene expression that may contribute to tumorigenesis [2]. CRFs are broadly classified in three main groups: histone tail modifiers (including histone acetyltransferases, histone deacetylases (HDACs), histone methyltransferases and histone demethylases, that deposit or remove acetyl or methyl groups, respectively); DNA methyltransferases (DNMTs) and putative demethylases (that affect cytosines at CpG islands); and ATP-dependent chromatin remodeling complexes (responsible for the repositioning of nucleosomes).

Until recently, DNMT proteins had not been found mutated in cancer [3], but DNMT3A, and later DNMT1 and DNMT3B, were reported as altered in patients with myelodysplastic syndromes and in acute monocytic leukemia, where their mutation status also predicted prognosis [4,5]. Mutations in ATP-dependent chromatin-remodeling complexes are recurrent in, amongst others, ovarian and clear cell renal cancers [2]. The regulation of the trimethylation of histone H3 at K27 mark (H3K27me3) by the Polycomb complex, a key component to maintain stem cell identity, is also frequently compromised in a variety of cancer types, including those from breast, bladder, pancreas, prostate and lymphomas [6]. Histone demethylases have also been implicated in the development of a wide variety of tumors. Moreover, recent whole exome sequencing studies in large cohorts of tumor samples have highlighted as main findings the inactivating mutations on proteins that regulate the epigenomic state of cells [7]. Alterations in KAT6B [8], SMARCC1 [9] and NSD1 [10] have been described in uterine, cervical and skin pre-malignant lesions, respectively. This presents these proteins as potential biomarkers, thus adding early cancer detection to the possible uses of CRFs in the clinic.

This current accumulation of evidence for the role of CRFs in cancer has attracted the attention of the scientific community towards CRFs as novel targets for cancer treatment. In 2006, the first HDAC inhibitor (HDACi), Vorinostat, was approved by the US Food and Drugs Administration to treat a specific type of lymphoma, and more than 20 molecules of this type are currently under preclinical and clinical investigation [11]. Some DNMT inhibitors have been recently approved by the US Food and Drugs Administration to treat myelodysplastic syndromes, and their combination with HDACi is a subject of intense study in clinical trials [12]. Some studies raise hopes for the possible use of HDACis to overcome drug resistance [13]. Interestingly, an in-depth review by Patel *et al*. on 46 potentially druggable yet chemically unexplored proteins in the Cancer Gene Census (CGC) identified six CRFs: ATRX, KAT6A, KDM6A, NSD3, PBRM1 and SMARCA4 [14].

Even though CRFs are emerging as important players in cancer development [15-20], to our knowledge no systematic analysis on the alterations of a comprehensive catalog of CRFs in different tumors has been performed to date. Moreover, most studies have focused their efforts in the in-depth characterization of specific genes that appear mutated at high frequencies, underestimating the impact of lowly recurrent drivers (those genes whose mutation is likely to be functional, but occurs in few samples) on tumorigenesis. For instance, a very recent report [21] focused only on the SWI/SNF family took into account the frequency of mutations of their members rather than their likelihood of driving tumorigenesis.

In this paper, we carry out a systematic exploration of the role of CRFs in tumorigenesis in different tissues. To that end, we first compiled and manually curated a comprehensive list of CRFs, for which we annotated any previously known implications in cancer. Secondly, we analyzed 4,623 tumor samples from 13 anatomical sites to identify which of the CRFs are driver candidates in these different sites, employing two approaches recently introduced by us [22,23]. Finally, we took advantage of the profiles of genomic and transcriptomic alterations revealed by the Cancer Cell Line Encyclopedia (CCLE) [24] to explore the effects of mutations in two likely driver CRFs on the expression of broad gene modules across 905 cancer cell lines.

## Results

### Analysis of chromatin regulatory factor tumor somatic mutations identifies 34 likely drivers in 13 cancer sites

To determine which CRFs may be involved in cancer emergence and development in primary tumors from 13 anatomical sites upon mutation, we first collected and manually curated a list of CRFs from the literature. This catalog contained 183 proteins grouped into eleven major functional classes, the most populated of which were the HDACs, the histone acetyltransferases and the histone methyltransferases. (The detailed list of CRFs in all functional classes is presented in Additional file 1: Table S1). Only 26 of them are included in the CGC. However, we found that many of these CRFs (115 out of 183) have some evidence, mainly in scattered reports from the past two years, of genomic or transcriptomic alterations in human tumors (Table 1 and Additional file 1: Table S2).

**Table 1:** Described oncogenic alterations in chromatin regulatory factors that are candidate drivers in at least one tissue

***Gene***	**Literature evidence**
***ARID1A***	Mutated in cc ovarian carcinoma and RCC (CGC), bladder [25], HCC [26], endometrium [27], colorectal [28], gastric adenocarcinoma [29], pancreatic cancer [30], lung adenocarcinoma [31], Burkitt lymphoma [32] and aggressive neuroblastoma [33].
	Down-regulated in aggressive breast cancer [34],
***KMT2C***	Mutated in medulloblastoma (CGC), HCC [26], bladder [25], prostate cancer [35], colorectal cancer [36], gastric adenocarcinoma [29], NSCLC [37], breast cancer [38] and pancreatic cancer [30].
	Deleted in leukemia [39].
***DNMT3A***	Mutated in AML (CGC), ALL and lung cancer [40].
	Over-expressed in ovarian aggressive tumors [41].
***KDM6A***	Mutated in kidney, esophageal squamous cell carcinoma, multiple myeloma (CGC), lung cancer [42], medulloblastoma [43], ccRCC [44], bladder [25] and prostate [35].
	Over-expressed in breast tumors with poor prognosis [45].
	Deleted in lung cancer [46].
***PBRM1***	Mutated in ccRCC, breast (CGC) and pancreatic cancer [47].
***NSD1***	Mutated in AML (CGC) and NMSC [10].
	Gained in lung adenocarcinoma of never-smokers [48].
***TET2***	Mutated in MDS (CGC), CMML and AML [49].
***SETD2***	Mutated in ccRCC (CGC).
	Down-regulated in breast tumors [50].
***SMARCA4***	Mutated in NSCLC (CGC), lung adenocarcinoma [31], medulloblastoma [43] and Burkitt lymphoma [32].
	Over-expressed in glioma [51] and in melanoma progression [52].
	Gained in lung [42].
***KMT2D***	Mutated in medulloblastoma, bladder [25], renal cancer (CGC), DLBCL [53].
	Over-expressed in breast and colon tumors [54].
*CHD4*	Mutated in high MSI gastric and colorectal cancers [55].
	Down-regulated in gastric and colorectal cancers [55].
*NCOR1*	Mutated in breast [56] and bladder cancer [25].
	Down-regulated in aggressive breast tumors [57].
***EP300***	Mutated in colorectal, breast and pancreatic cancers, ALL, AML, DLBCL (CGC), bladder [25], SCLC [58] and endometrium [27].
	Up-regulated in esophageal squamous cell carcinoma [59] and advanced HCC [60].
	Loss of heterozygosity in glioblastoma [61].
***KDM5C***	Mutated in ccRCC (CGC).
***ARID2***	Mutated in hepatocellular carcinoma (CGC), melanoma [62], NSCLC [63] and pancreatic cancer [30].
	Deleted in NSCLC [63].
*ATF7IP*	-
***ASXL1***	Mutated in MDS and CMML (CGC), myeloproliferative neoplasm; [64], AML with myelodysplasia-related changes [65] and castration-resistant prostate cancer [66].
***MLL***	Mutated in AML, ALL (CGC), bladder [25], SCLC [58], HCC [26] and gastric tumors [29].
*BAZ2A*	Over-expressed in CLL [67].
*CHD3*	Mutated in high MSI gastric and colorectal cancers [55].
***ATRX***	Mutated in pediatric glioblastoma, neuroendocrine pancreatic tumors (CGC) and high grade adult gliomas [68].
*ARID1B*	Mutated in breast tumors [56].
*MBD1*	Over-expressed in pancreatic cancer [69].
***BAP1***	Mutated in uveal melanoma, breast, NSCLC and RCC (CGC).
	Over-expressed in NSCLC with good prognosis [70].
*INO80*	-
*CHD2*	Mutated in high MSI gastric and colorectal cancers [55] and CLL [71].
	Down-regulated in relapsed colon cancer [72].
*ARID4A*	-
*DOT1L*	-
*ASH1L*	Mutated in lung cancer cell lines [42].
	Gained in hepatocellular carcinoma [73].
*BPTF*	Gained in neuroblastoma and lung cancer [74].
*RTF1*	-
*PHC3*	Mutated and lost in osteosarcoma [75].
*SMARCA2*	Mutated in NMSC [76] and CLL [77].
	Down-regulated in lung adenocarcinoma [78] and gastric cancer [79].
	Amplified in AML [80].
*SETDB1*	Recurrently amplified and over-expressed in melanoma [81].

**Figure 1:**
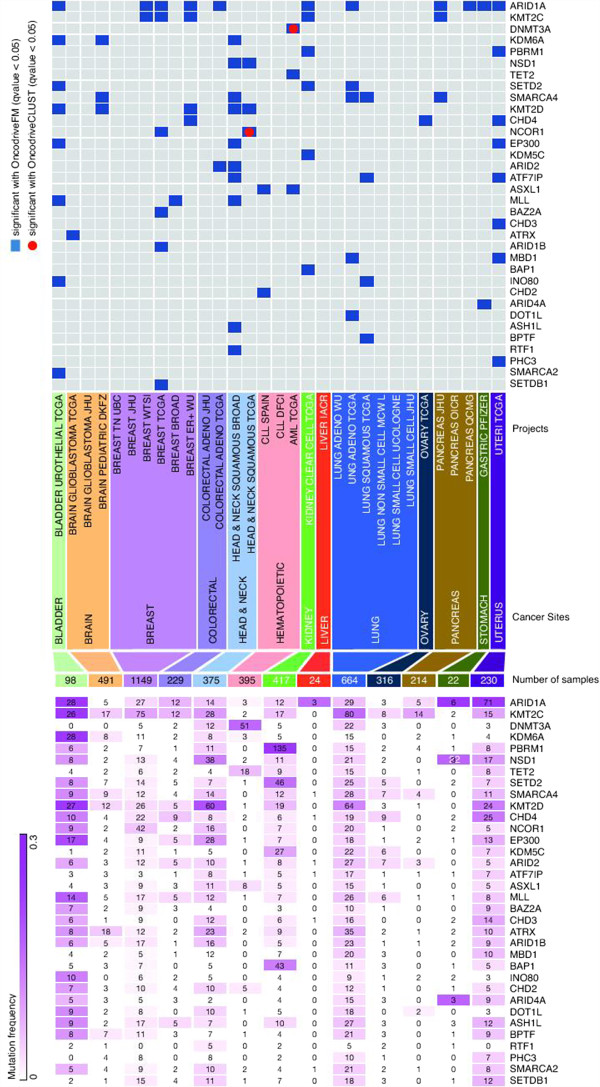
**Likely driver chromatin regulatory factors across the datasets of somatic mutations in IntOGen-mutations.** The heat-map in the top panel identifies FM-biased and CLUST-biased CRFs across the 31 datasets from 13 sites in IntOGen-mutations, whose original projects are detailed in the middle panel. The heat-map in the bottom panel contains the number of samples with mutations in each likely driver CRF in each site. Cells in the heat-map are colored following mutational frequency.

This is an exhaustive compilation of alterations (^a^) reported in CRFs showing FM bias and CLUST bias in at least one tissue (Figure 1). Gene names correspond to HUGO Gene Nomenclature Committee-approved symbols. In bold typeface, genes included in the CGC [82]. ALL, acute lymphocytic leukemia; AML, acute myeloid leukemia; cc, clear cell; CGC, Cancer Gene Census; CLL, chronic lymphocytic leukemia; RCC, renal cell carcinoma; CMML, chronic myelomonocytic leukemia; CRPC, castration-resistant prostate cancer; ESCC, esophageal squamous cell carcinoma; HCC, hepatocellular carcinoma; HL, Hodgkin lymphoma; MCL, mantle cell lymphoma; MDS, myelodysplastic syndrome; MSI, microsatellite instability; MPN, myeloproliferative neoplasm; NMSC, non-melanoma skin cancer; NSCLC, non-small cell lung carcinoma; RCC, renal cell carcinoma.

^a^Evidence based solely on cancer cell lines is excluded from this table. Only evidence in human samples have been used. Effects of pharmacological inhibition are not included. Germline polymorphisms are also excluded.

In IntOGen-mutations [83], during the past year, we have collected and analyzed datasets of cancer somatic mutations produced by several research groups across the world. Some of them have been generated within the framework of large international initiatives like The Cancer Genome Atlas (TCGA) [84] and the International Cancer Genomes Consortium [85], while others are the fruit of independent laboratories. Taken together, these datasets [86] contain somatic mutations detected in 4,623 primary tumor samples obtained from 13 anatomical sites (Table 2). Each dataset has been analyzed separately, to compensate for differences between tumor histologies and subtypes, and between sequencing analysis pipelines. First, we used an approach recently developed by us, OncodriveFM [22], to detect genes that, across the cohort of tumor samples, tend to accumulate functional mutations. We give the name 'FM bias’ to this significant trend towards the accumulation of functional mutations. The FM bias is a signal of positive selection during cancer development and therefore FM-biased genes are likely candidates to drivers. Second, we identified genes whose mutations tend to significantly cluster in certain regions of their protein sequence (CLUST bias) also via an approach recently developed in our group, OncodriveCLUST [23]. Both FM-biased and CLUST-biased genes constitute sound candidates to cancer drivers [87] in these 13 anatomical sites. We have also combined the *P* values of FM bias and CLUST bias of individual genes across the datasets of tumor samples obtained from the same anatomical site. In summary, we have obtained a measurement of FM bias and CLUST bias for each mutated gene at the level of one dataset of tumor samples (or project), and also at the level of each anatomical site (or tissue). This catalog of likely driver genes has allowed us, for the first time, to systematically explore the involvement of epigenetic mechanisms (via mutations in CRFs) in tumorigenesis in 4,623 tumor samples from 13 anatomical sites.

**Table 2:** Description of the datasets of tumor somatic mutations collected and analyzed to detect candidate cancer driver genes

**Site**	**Dataset name**	**Description**	**Authors**	**Obtained from**	**Tumor samples**	**References**
**Bladder**	BLADDER UROTHELIAL TCGA	Bladder urothelial carcinoma	TCGA	Synapse	98	-
**Brain**	BRAIN GLIOBASTOMA TCGA	Glioblastoma multiforme	TCGA	Synapse	290	[84]
	BRAIN GLIOBASTOMA JHU	Glioblastoma multiforme	John Hopkins University	ICGC DCC	88	[88]
	BRAIN PEDIATRIC DKFZ	Pediatric brain tumors	DKFZ	ICGC DCC	113	[89,90]
**Breast**	BREAST JHU	Breast cancer	Johns Hopkins University	ICGC DCC	42	[91]
	BREAST WTSI	Breast cancer	Welcome Trust/ Sanger Institute	ICGC DCC	100	[56]
	BREAST TN UBC	Triple negative breast cancer	University of British Columbia	PubMed	65	[92]
	BREAST TCGA	Breast invasive carcinoma	TCGA	Synapse	762	[93]
	BREAST BROAD	Breast cancer	BROAD Institute	PubMed	103	[94]
	BREAST ER + WU	ER + breast cancer	Washington University	PubMed	77	[38]
**Colorectal**	COLORECTAL ADENO JHU	Colorectal adenocarcinoma	Johns Hopkins University	ICGC DCC	36	[91]
	COLORECTAL ADENO TCGA	Colorectal adenocarcinoma	TCGA	Synapse	193	[28]
**Head and neck**	HEAD/NECK SQUAMOUS BROAD	Head and neck squamous cell carcinoma	Broad Institute	SM	74	[95]
	HEAD/NECK SQUAMOUS TCGA	Head and neck squamous cell carcinoma	TCGA	Synapse	301	-
**Hematopo-ietic**	CLL SPAIN	Chronic lymphocytic leukemia	Spanish Ministry of Science	ICGC DCC	109	[71,96]
	CLL DFCI	Chronic lymphocytic leukemia	Dana Farber Cancer Institute	SM	90	[97]
	AML TCGA	Acute myeloid leukemia	TCGA	Synapse	196	[98]
**Kidney**	KIDNEY CLEAR CELL TCGA	Kidney clear cell carcinoma	TCGA	Synapse	417	[99]
**Liver**	LIVER IARC	Liver cancer	IACR	ICGC DCC	24	[100]
**Lung**	LUNG ADENO WU	Lung adenocarcinoma	Washington University School of Medicine	ICGC DCC	162	[101]
	LUNG NON SMALL CELL MCW	Non small cell lung cancer	Medical College of Wisconsin	SM	31	[37]
	LUNG SQUAMOUS TCGA	Lung squamous cell carcinoma	TCGA	Synapse	174	[102]
	LUNG ADENO TCGA	Lung adenocarcinoma	TCGA	Synapse	228	-
	LUNG SMALL CELL UCOLOGNE	Small cell lung cancer	University Cologne	SM	27	[58]
	LUNG SMALL CELL JHU	Small cell lung cancer	Johns Hopkins University	SM	42	[103]
**Ovary**	OVARY TCGA	Ovarian serous cystadenocarcinoma	TCGA	Synapse	316	[104]
**Pancreas**	PANCREAS JHU	Pancreatic cancer	Johns Hopkins University	ICGC DCC	114	[105]
	PANCREAS OICR	Pancreatic cancer	Ontario Institute for Cancer Research	ICGC DCC	33	[106]
	PANCREAS QCMG	Pancreatic cancer	Queensland Centre for Medical Genomics	ICGC DCC	67	[106]
**Stomach**	GASTRIC PFIZER	Gastric cancer	Pfizer Worldwide Research and Development	SM	22	[107]
**Uterus**	UTERI TCGA	Uterine corpus endometrioid carcinoma	TCGA	Synapse	230	-

The results of all the analyses may be browsed and retrieved through IntOGen-mutations. TCGA, The Cancer Genome Atlas; ICGC, International Cancer Genomes Consortium; DCC, ICGC Data Coordination Center; DKFZ, German Cancer Research Center; IACR, International Agency for Research on Cancer; SM, Supplementary Material of articles.

**Figure 2:**
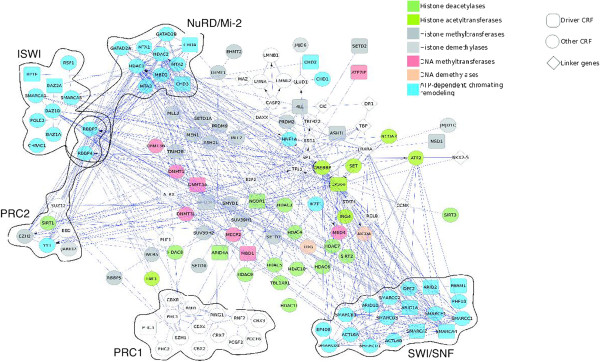
**Chromatin regulatory factors within their context of functional interactions.** Network of functional interactions among CRFs mapped to the Cytoscape FI plugin network. Square nodes represent likely driver CRFs, circle nodes other CRFs within the catalog, and diamond nodes represent linker genes. CRFs functions are color-coded, and genes in the same complex are grouped and circled.

After an exhaustive search within the list of likely driver genes, we found that 34 CRFs from our manually curated list are FM biased and/or CLUST biased in at least one site (Figure 1, upper panel). Sixteen of them appear as likely drivers in more than one project, and only liver carcinomas appear free of likely driver CRFs - although this may be attributed to the small sample size of the dataset. Several driver CRFs are mutated at frequencies above 10% in at least one site (Figure 1, lower panel). Functional relationships among many of them - 124 CRF genes can be mapped onto a pre-compiled [108] functional interaction network (Figure 2) - suggest the possibility that mutations in different genes produce similar malignancies (see below). We can therefore make the general observation that CRFs - 34 in the dataset collected by us - potentially act as mutational drivers in most of the cancer sites studied.

**Figure 3:**
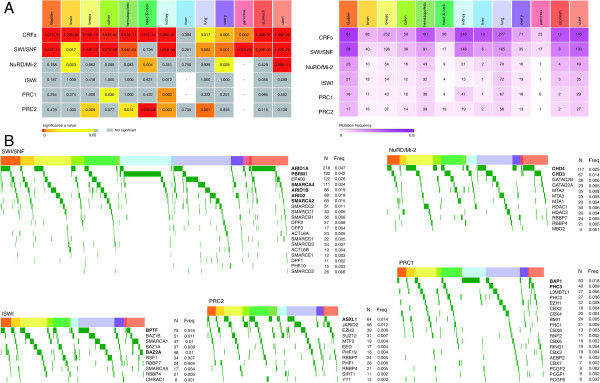
**FM bias, mutation frequencies and mutually exclusivity of chromatin regulatory factors as part of complexes. (A)** Left heat-map shows the *P* value of FM bias analysis for all CRFs and for each complex. Right heat-map shows the number of samples with PAMs in the complex and the color indicates the mutation frequency (number of samples with PAMs divided by number of samples of this cancer type analyzed). **(B)** Heat-map of samples and genes of each complex, PAMs are represented as green cells in the heat-map. Tumor samples from each site have headers of the corresponding colors. Samples and genes in the heat-map are ordered based on mutually exclusive alterations within each site using Gitools built-in function for this purpose. Number of samples with PAMs in the gene (N) and the mutation frequency (Freq) of the gene in whole dataset are shown at the right of each heat-map. Gene names in bold indicated that the gene is one of the 34 detected as candidate drivers. PAM, protein-affecting mutations.

Driver candidates are significantly overrepresented within our catalog of CRFs (34 driver CRFs from 183 human CRFs in our list versus 348 total drivers from 22,696 human genes; Fisher’s *P* value 1.26 × 10^-25^). In addition, when analyzed as a group, the 183 CRFs in our catalog appear FM biased in all sites except liver (Figure 3A), which indicates that collectively they tend to accumulate mutations that on average possess higher functional impact than the background of the corresponding tumors. Taken together, these two observations suggest that CRFs as a group may have an important role in tumorigenesis in the 13 sites with data in IntOGen.

Because CRFs usually act as multiprotein complexes, we also determined whether the best established of these complexes exhibit discernible signals of positive selection as a group across tumor samples. We did this in two ways. First, we computed the FM bias of six complexes described in Additional file 1: Table S1 and whose components appear illustrated in the network of functional interactions in Figure 2. We established that five of the complexes - ISWI being the exception - significantly accumulate highly impacting mutations in at least one site (Figure 3A). Second, we observed that pairs of proteins of the same complex tend to be mutated following a pattern of mutual exclusivity within cancer sites (Figure 3B and Additional file 1: Table S3). For example, the exploration of the SWI/SNF complex in breast tumors revealed that *ARID1A* tends to be mutated in samples where neither *SMARCA4*, *ARID2* nor *SMARCA2* are mutated. These two observations imply that multiprotein complexes, rather that individual genes, are the subjects of positive selection during tumorigenesis in the cancer sites under study.

### The implication of chromatin regulatory factors in tumorigenesis strongly depends on the anatomical site and the tumor type

**Figure 4:**
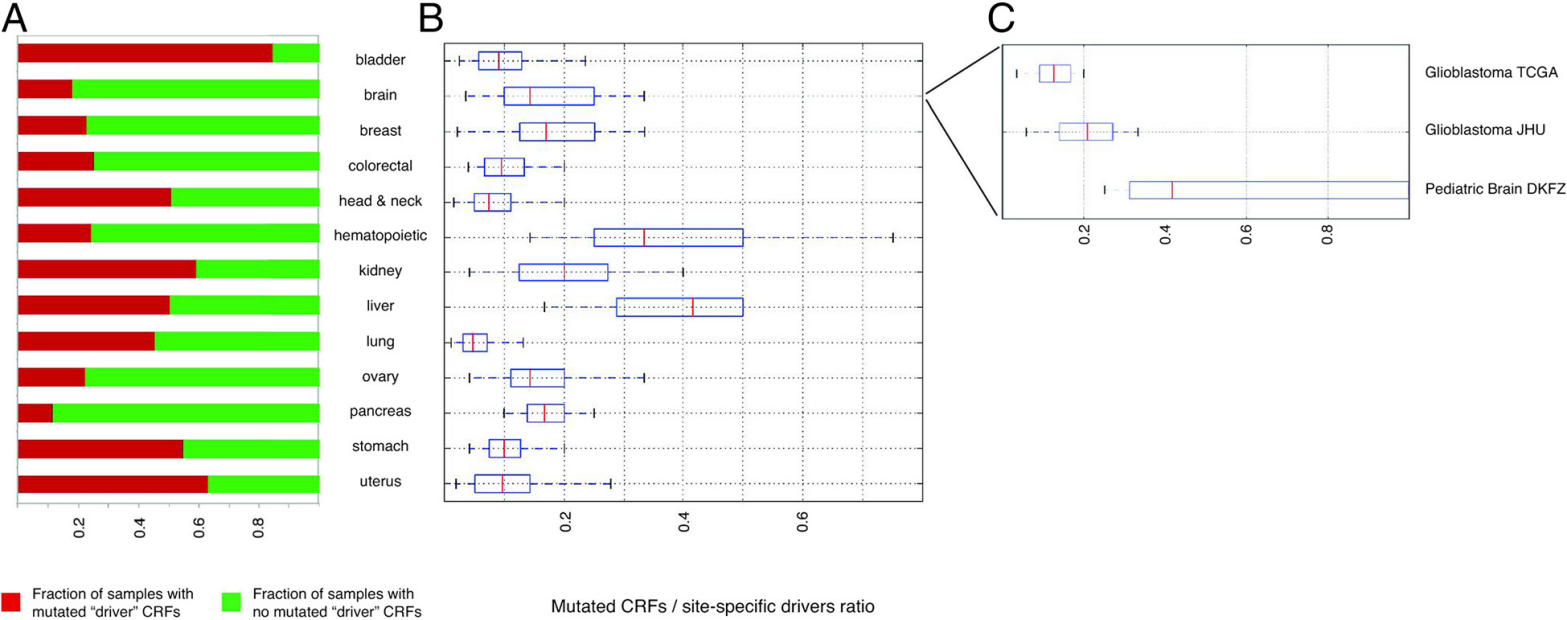
**Relative importance of chromatin regulatory factors in tumorigenesis across sites. (A)** Histograms of the fraction of samples with 0 (green) or at least one (red) likely driver CRF with PAMs in each site. **(B)** Boxplots representing the distribution of fraction of CRFs with PAMs with respect to all FM-biased genes with PAMs in each sample (CF ratios) of samples from each site with at least one mutation in a CRF (red fraction in panel A). **(C)** Boxplots representing the distribution of CF ratios of samples from each of the three projects focused on brain tumors. CRF, chromatin regulatory factors; DKFZ, German Cancer Research Center; JHU, Johns Hopkins University; TCGA, The Cancer Genome Atlas.

To determine whether there are differences in the implication of CRFs in tumorigenesis across the anatomical sites in IntOGen, we first computed the number of likely driver genes in general, and likely driver CRFs in particular, that bear protein sequence-affecting mutations, or PAMs (non-synonymous, stop, frameshift-causing insertions or deletions (indels)) in each tumor sample. From these data, the simplest way of representing the relative importance of mutations in CRFs in tumorigenesis across sites consists of counting the number of samples with at least one FM-biased CRF bearing a PAM (Figure 4A). In this metric, bladder urothelial carcinomas and endometrial carcinomas stand out, with more than 80% and 60%, respectively, of the samples with at least one mutated CRF. On the opposite extreme, less than 10% of brain and hematopoietic tumor samples contain mutated likely driver CRFs.

**Figure 5:**
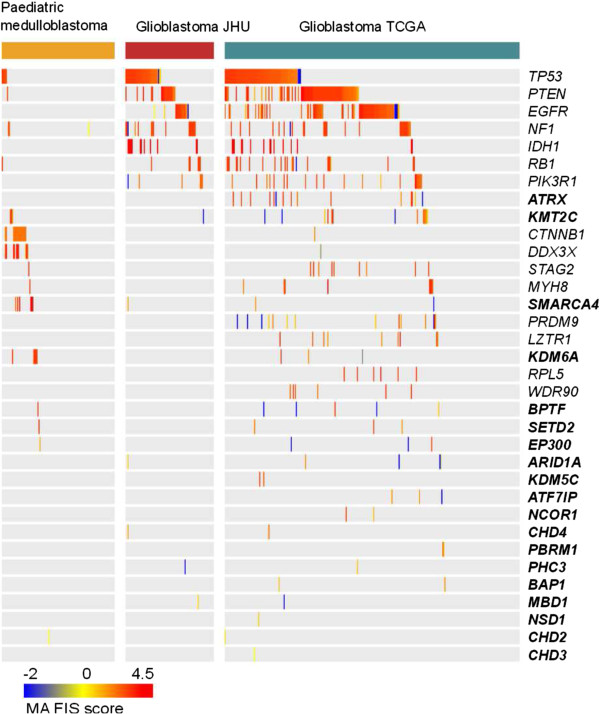
**Mutational status of tumor samples from the three brain datasets included in IntOGen.** The genes represented in the heat-map comprise all FM-biased CRFs that bear one mutation in at least one brain tumor sample (in bold typeface) plus the top 15 FM-biased genes in brain obtained from IntOGen. Mutations are represented by their MutationAssesor [109] functional impact scores (FIS). Samples and genes in the heat-map are ordered based on mutually exclusive alterations within dataset. FIS, functional impact score; MA, MutationAssessor score. JHU, Johns Hopkins University; TCGA, The Cancer Genome Atlas.

We then computed the fraction of CRFs with PAMs with respect to all FM-biased genes with PAMs in each sample (CF ratio) (Figure 4B). This measure gives an indication of the relative importance of CRFs in the tumorigenesis process in each sample. Although liver or hematopoietic are not among the sites with the highest proportion of tumor samples with mutated CRFs (Figure 4A), these appear to be very important in the development of tumors in these sites (see the corresponding boxplots of Figure 4B). A closer look at the repertoire of mutated drivers in the samples of the three brain tumor datasets currently in IntOGen reveals that whereas mutations in classic tumor suppressors and oncogenes dominate the landscape of glioblastomas, mutations in CRFs are more predominant in pediatric medulloblastomas. The median of the ratio of mutations in CRFs to mutations in all drivers across medulloblastoma samples is 0.4, compared to 0.21 and 0.1 in glioblastoma JHU (Johns Hopkins University; see Table 2) and glioblastoma TCGA (The Cancer Genome Atlas; see Table 2), respectively (Figure 4C). The samples of these two glioblastoma datasets exhibit a repertoire of mutated 'classical’ tumor suppressors and oncogenes, such as *TP53*, *PTEN* and *EGFR* (Figure 5). As observed in the previous section, mutations in CRFs are likely drivers in tumors from most cancer types. Nevertheless, the latter result suggests that these mutations are circumscribed to a relatively small number of tumor samples, although future reviews of the catalogs of CRFs may increase the proportions calculated here.

### Mutations in chromatin regulatory factors correlate with transcriptomic alterations of gene modules in cancer cell lines

To further understand the possible implication of CRFs in tumorigenesis, we explored the effects of CRF mutations on changes in the transcriptional levels of broad gene sets in cancer cell lines. To this end, we employed the data produced by The Cancer Cell Line Encyclopedia project, which has sequenced 1,651 protein-coding genes, of which 43 are CRFs according to our curated list (see Additional file 1: Table S1 for a detailed classification). First, to check whether cancer cell lines behave comparably to primary tumors in the patterns of altered gene modules, we carried out a sample level enrichment analysis (SLEA) [110] over cancer cell lines using Gene Ontology Biological Process terms that are altered in specific cancer tissues. We found that cancer cell lines repeated the transcriptional profiles typical of their corresponding primary tumors (Additional file 2: Figure S1).

**Figure 6:**
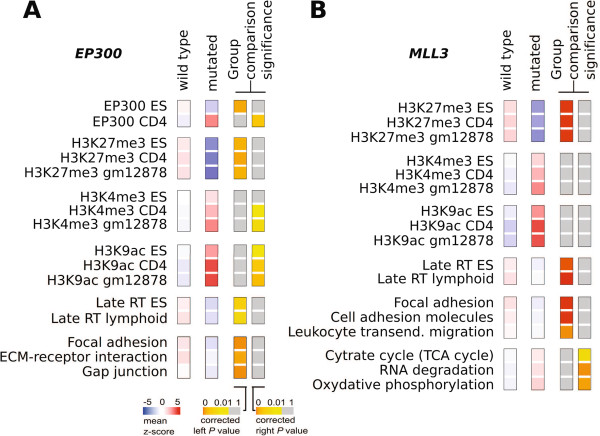
**Effect of PAMs in *EP300 *and *MLL3 *on the transcription of broad gene modules across cancer cell lines.** Cancer cell lines originated from solid tissues (Additional file 2: Figure S1) are enriched (SLEA) for regulatory modules (Additional file 1: Table S4) and selected pathways from Kyoto Encyclopedia of Genes and Genomes. The first two panels in both A and B correspond to mean enrichment z-scores in wild type and mutant cell lines. The difference between the two enrichment groups, assessed through a Wilcoxon-Mann–Whitney group comparison test, is indicated at the right. **(A)***EP300* mutation status. **(B)***MLL3* mutation status.

We then assessed the transcriptional impact of PAMs on *EP300* and *MLL3* (the only CRFs sustaining PAMs in sufficient cell lines: 115 and 191, respectively) to determine whether the impact of these PAMs on epigenetic regulation could translate into changes of the transcriptional levels of broad gene sets. The underlying hypothesis was that genes whose transcription was modulated by specific histone marks that became affected by PAMs on these two genes would present expression changes detectable when analyzed as a group. We collected regulatory modules of histone modifications in three cell types (Additional file 1: Table S4) and performed SLEA separately on cell lines originated from blood and solid tissues (Figure 6). As a result of the SLEA, we obtained a value of significance of the over-expression or under-expression (as a z-score) of each module in each cell line. We then compared the z-scores of cell lines that bear mutations in the gene in question (*EP300* or *MLL3*) to those cell lines where it does not, using the Wilcoxon-Mann–Whitney test. The *P*-values of the right-tail and left-tail comparisons were then adjusted using the Benjamini-Hochberg approach. Figure 6 presents the modules that rendered either significant right-tail or left-tail *P* values for any of the two genes. It shows that, in general, cell lines from solid tissues with mutations in either *EP300* or *MLL3* exhibited lower expression of repressed chromatin gene modules (H3K27me3 and late replicating genes), and higher expression of gene modules with activating histone marks (marked by H3K4me3 and H3K9ac; Table 1). The under-expression of the H3K27me3 module, regulated by Polycomb, has been associated to a stem cell-like signature and more aggressive tumors [86]. Moreover, cell lines with mutations in *MLL3* showed higher expression of cell cycle-related modules. Taken together, these results suggest that mutations in CRFs may affect the transcriptional levels of gene sets bearing histone marks related to these CRFs.

## Discussion

In this study, we found that several CRFs are likely involved in tumorigenesis in cancers from 13 anatomical sites. We uncovered these genes as putative causes of the studied malignancies through the use of the FM bias and CLUST bias analyses, rather than the mere recurrence of mutations in genes across tumor samples. Moreover, by focusing on multiprotein complexes formed by several CRFs, we found evidence that suggest that these, rather than individual genes, are the subjects of positive selection during tumorigenesis. These two approaches constitute novelties with respect to the most recent and comprehensive analysis [21], which found recurrent mutations in SWI/SNF proteins across more than 650 tumor samples of 10 anatomical sites. Another important methodological novelty of our work consists in the use of CF ratios to assess the relevance of mutations in CRFs in tumorigenesis in cancers from different sites. The employment of this ratio normalizes the number of CRFs with PAMs in the samples of a site by its intrinsic burden of mutations in driver genes. It is thus possible to observe that PAMs in CRFs, although highly prevalent in carcinomas of the uterus, probably play a relatively small role in their tumorigenesis because these tumors bear mutations in many other driver genes. However, mutations in CRFs appear to play a bigger role in tumorigenesis in hematopoietic malignancies than they do in tumors from other sites, although only few hematopoietic tumors bear PAMs in CRFs (Figure 4).

A group of pediatric medulloblastomas also possess abnormally high CF ratios, which implies that a high proportion of their mutated drivers are actually CRFs. It has been suggested that both pediatric and hematopoietic malignancies have very low mutational rates and therefore fewer drivers take part in their emergence than in solid adult tumors [111]. One could hypothesize from our results that alteration of either the transcriptional control or the chromatin maintenance of broad gene modules - as we observed in cell lines - via mutations in CRFs may be the crucial step of tumorigenesis in at least some of these tumors. This hypothesis, which could be experimentally tested, is another important contribution of the present work.

A third important contribution is the list of putative driver CRFs, which is available at IntOGen [112]. In particular, two of them were uncovered as putative drivers in more than one site (*CHD4* and *ATF7IP*) and are not annotated in the CGC [82]. They therefore constitute interesting candidates for novel epigenetic drivers (Figure 1). These additions to the list of driver CRFs might contribute to the research for anticancer drugs that takes CRFs as suitable targets.

## Conclusions

We present the first systematic approach to characterize the repertoire of CRFs that could constitute mutational cancer drivers in tumors from 13 anatomical sites. We found that likely driver CRFs appear across tumor samples from most of these 13 sites, although the number of affected samples is in general low, except in the case of tumors from several sites, such as bladder, kidney and uterus. Mutations in CRFs appear to be in general only one of several contributing mechanisms towards tumorigenesis in most cancer samples. Finally, we have proved that mutations in two CRFs correlate with broad expression changes across cancer cell lines, thus presenting at least one mechanism through which these mutations could contribute to tumorigenesis in cells of the corresponding tissues. Our results expand the current knowledge on the involvement of CRFs in tumorigenesis in several malignancies. Furthermore, they can contribute to formulate hypotheses on the mechanistic basis for this association. All the results presented here are available for browsing through the IntOGen-mutations platform [83,112] and using Gitools interactive heat-maps [113].

## Materials and methods

### Chromatin regulatory factors

We manually compiled a list of 183 genes coding for CRF proteins from the literature, based on protein function and known essential association to complexes important for the regulation of chromatin structure. A detailed classification of these CRFs is presented in Additional file 1: Table S1; the information was obtained from the Uniprot database [114] and the manuscripts referenced within the additional file. The relevant proteins for the purpose of this analysis are described in Table 1 and Additional file 1: Table S2.

### FM-biased genes in primary tumors

FM-biased genes exhibit a bias towards the accumulation of functional mutations across a cohort of tumor samples and are therefore candidate cancer drivers. We have compiled 31 datasets of tumors from 13 anatomical sites and detected the FM-biased genes in each of them with the approach described in [22]. Genes that were not expressed across the major (TCGA) datasets included in IntOGen (obtained from syn1734155) were eliminated from the OncodriveFM analysis at this point. The overlap of drivers obtained from different datasets of mutations detected in tumors from the same anatomical site is shown in Additional file 2: Figure S2. Finally, we combined the gene-wise *P* values obtained for datasets of the same anatomical site to obtain a single *P* value that measures the bias of the gene towards the accumulation of functional mutations in different tumors from the same site. The corrected genes FM bias *P* values in these 13 tissues are stored in the IntOGen knowledgebase [87]. The collection of the datasets of tumor somatic mutations, their processing and browsing through IntOGen are thoroughly described in [83]. Details of the 31 tumor somatic mutations datasets are presented in Table 2.

### CLUST-biased genes in primary tumors

PAMs in CLUST-biased genes tend to be grouped in regions of the proteins in a higher degree than synonymous mutations across the same dataset. This grouping constitutes another signal of positive selection that points to likely cancer drivers. The method to compute the CLUST bias in genes across datasets of tumor somatic mutations (OncodriveCLUST) is described in Tamborero *et al*., [23]. We computed the CLUST bias of all genes with PAMs across the 31 datasets compiled and stored in IntOGen-mutations [83]. Genes that were not expressed across the major (TCGA) datasets included in IntOGen (obtained from syn1734155) were eliminated from the OncodriveCLUST analysis at this point. As with the FM bias, we combined the gene-wise *P* values obtained for datasets of tumor samples from the same anatomical site. The corrected genes’ CLUST bias *P* values in these 13 tissues are stored in the IntOGen knowledgebase [87]. The collection of the datasets of tumor somatic mutations, their processing and browsing through IntOGen are thoroughly described in [83]. Details of the 31 tumor somatic mutations datasets are presented in Table 2.

### Analysis of mutational frequencies of tumor samples

We defined a group of broad consequence types as corresponding to PAMs for all analyses of the mutational frequencies of tumor samples. All non-synonymous, stop and frameshift indels were included in this group. We recorded two numbers in the 4,623 tumor samples included in the study: the number of PAMs in any of the 34 likely driver CRFs detected across the 13 sites; and the number of PAMs in any of the 382 likely driver genes detected across the 13 sites. We then computed the ratio (CRFs-to-drivers ratio, or CF ratio) between these two numbers to assess the relevance of mutations in CRFs in tumorigenesis in every tumor sample. Note that because the 34 likely driver CRFs were included within the catalog of 382 likely drivers, the CF ratio takes values between 0 (no mutations in CRFs) and 1 (all mutated drivers in the sample are CRFs). Finally, we computed the number of tumor samples from each site with at least one PAM in a CRF and the distribution of their CF ratios.

### Functional network analysis

We mapped the 183 CRFs in our catalog to the functional interactions network within the Cytoscape FI plugin [108,115], allowing the presence of linker genes to maximize the number of connected CRFs. Using Cytoscape, we then grouped genes in the same multiprotein complex (from the ones shown in Additional file 1: Table S2). We also mapped the biological functions of CRFs in the network using nodes colors, and whether they appeared as likely drivers through nodes shapes.

### Cancer cell lines data processing

Expression arrays from the CCLE were downloaded from the Gene Expression Omnibus [GEO:GSE36133] as raw CEL files, and pre-processed as previously described [110]. The input data for enrichment analysis was obtained by median centering the expression value of each gene across cancer cell lines and dividing this value by the standard deviation. The obtained value is the measure of expression level for the gene in a sample as compared to its expression level in all other samples in the dataset. We built separate expression matrices for cancer cell lines obtained from hematological system or solid primary cells, since the expression profiles of these two groups were shown to clearly differ in the original publication [24].

SLEA was performed using Gitools version 1.6.0 [116]. We used the z-score method as described previously [117]. This method compares the mean (or median) expression value of genes in each module to a distribution of mean (or median) of 10,000 random modules of the same size. Such enrichment analysis is run for each sample and the result is a z-score, which is a measure of the difference between the observed and expected mean (or median) expression values for genes in a module. We applied the mean z-score enrichment values, which are the arithmetic means of z-scores for individual samples, separately in cell lines obtained from hematological system or in those obtained from solid primary cells. To test for significant differences between the z-score means between groups of cell lines we used the Mann–Whitney test [118] implemented in Gitools. All heat-maps were generated with Gitools [119].

To detect potential PAMs in genes within the list of CRFs (Additional file 1: Table S1), we downloaded processed mutations data (single nucleotide variants and small indels) for 1,651 protein-coding genes (7 May 2012 version, excluding common polymorphisms and single nucleotide variants with an allelic fraction >10%) from the CCLE website [120]. We computed the consequence types of these variants using the Ensembl (v69) Variant Effect Predictor wrapped within the IntOGen-mutations pipeline [83].

### Public gene regulation datasets

We collected lists of genes occupied by a specific histone mark or bound by a regulatory factor, and computationally predicted chromatin states, from available sources (Additional file 1: Table S4). These included human genome-wide occupancy datasets from ChIP-seq experiments in several cell types [121-125] that we processed using Bowtie [126] (version 0.12.5, hg19 genome assembly, unique alignments, allowing two mismatches) for short read aligning. For peak detection of transcription factors we used MACS [127] (version 1.4.1, settings: --nomodel and --bw parameter set to twice the shift size whenever a control immunoprecipitation was not available). For broad histone modifications (that is, H3K27me3), we used SICER [128] (version 1.1, setting gap size to 600). Regions were assigned to protein-coding genes (Ensembl v69) if they overlapped either to the gene body or up to 5 kb upstream from the transcription start site, using BedTools [129]. Overall peak calling performance was evaluated with CEAS [130]. Other gene sets were obtained from KEGG [131] and Gene Ontology [132]. The list and mappings (in Ensembl v67 IDs) of KEGG and Gene Ontology Biological Process terms were downloaded through the Gitools importer [116].

## Abbreviations

CCLE: Cancer cell line encyclopedia; CF: Mutations in CRFs-to-mutations in drivers ratio; CGC: Cancer gene census; CRF: Chromatin regulatory factors; DCC: ICGC Data coordination center; DKFZ: German cancer research center; DNMT: DNA methyltransferases; H3K27me3: Trimethylation of histone H3 at K27 mark; HDAC: Histone deacetylases; HDACi: Histone deacetylase inhibitor; IACR: International Agency for Research on Cancer; ICGC: International cancer genomes consortium; JHU: Johns Hopkins University; PAM: Protein-affecting mutation; SLEA: Sample level enrichment analysis; SM: Supplementary Material of articles; TCGA: The cancer genome Atlas.

## Competing interests

The authors declare that they have no competing interests.

## Authors’ contributions

The three authors designed the study. AJ-S curated the list of CRFs and performed SLEA and analyses of mutations in cell lines. AG-P analyzed mutation data in 4,623 primary tumors and identified FM-biased and CLUST-biased genes in different tissues. NL-B supervised the study. The three authors contributed to drafting and editing the manuscript. All authors read and approved the final manuscript.

## Supplementary Material

Additional file 1 Supplementary Tables S1, S2, S3 and S4 with titles and descriptions, and supplementary references Click here for file

Additional file 2 Supplementary Figure S1 and S2. Click here for file

Additional file 3 Supplementary Table S5. Click here for file
