# Supplementary material for: The mutational landscape of chromatin regulatory factors across 4,623 tumor samples
Source: Genome Biol. 2013 Sep 24;14(9):r106. doi: 10.1186/gb-2013-14-9-r106 (PMC4054018; doi:10.1186/gb-2013-14-9-r106)
Supplement: Additional file 1 — Supplementary Tables S1, S2, S3 and S4 with titles and descriptions, and supplementary references [file gb-2013-14-9-r106-S1.pdf]

## *Supplementary tables*

### Table S1. Classification of Chromatin Regulatory Factors.

| Gene name(a) | Description | Function(b) |
|---|---|---|
| **Polycomb Repressive Complex 2** | | |
| *EZH2** | Catalytic subunit | H3K27me1/me2/me3 HMT. Major role in stem cell identity maintenance. Also methylates GATA4. Interacts with DNMTs. |
| *SUZ12* | EZH2 coenzyme | Required for PRC2 H3K27 HMT activity (1). Interacts with SIRT1. |
| *EED* | | Different isoforms determine PRC3 or PRC4 PRC2 variants. |
| *RBBP4 (RBAP46)** | | Required for the association of PRC2 to the histone tail (2). Binds Rb to regulate cell proliferation. |
| *RBBP7 (RBAP48)** | | Interacts with BRCA1 and may regulate cell proliferation and differentiation. |
| *PHF1 (PCL1)* | | Mediates PRC2 intrusion into active H3K36 chromatin regions (3). |
| *PHF19 (PCL3)* | | Mediates interaction of PRC2 with H3K36me3, essential for full PRC2 activity (4). |
| *ASXL1* | | Associates with PRC2 to promote gene repression (5). |
| *MTF2 (PCL2)* | | Required for PRC2-mediated Hox repression (6). |
| *JARID2 (JMJ)** | | Essential in embryonic development, inhibits H3K27me3 by PRC2. |
| *YY1** | | Interacts with PRC2, and it is required for EZH2-mediated H3K27me3 (7). Also part of chromatin remodelling INO80 complex. |
| *SIRT1** | Class III HDAC | Transiently interacts with PRC2. Histone and protein deacetylase activity. |
| **Polycomb Repressive Complex 1** | | |
| *EZH1* | Catalytic subunit | H3K27me1/me2/me3 HMT. Less critical for H3K27me3 formation than *EZH2*. |
| *BAP1* | | Catalytic component of the PR-DUB complex, that specifically deubiquitinates H2AK119ub1. |
| *BMI1* | | Maintenance of transcriptional repression of key genes during development. H2AK119ub. |
| *RING1* | | H2AK119ub. |
| *RNF2 (RING1B)* | | H2AK119ub. Acts as the main ub ligase in PRC1. |
| *CBX2* | | |
| *CBX3* | | Part of PRC1-like complex 4 (8). Binds the nuclear lamina through lamin B receptor. |
| *CBX4* | | |
| *CBX6* | | |
| *CBX7* | | Promotes H3K9me3. Regulates cellular lifespan by repressing CDKN2A. |
| *CBX8* | | |
| *PCGF1 (NSPC1)* | BCOR complex | Represses CDKN1A expression in a RARE-dependent manner. |
| *PCGF2 (MEL18)* | | |
| *PCGF6 (MBLR)* | | |
| *PHC1* | | |
| *PHC2* | | |
| *PHC3* | | |
| *AEBP2* | | |
| *L3MBTL1* | | Specifically recognizes me1 and me2 lysines. |
| **Histone deacetylases** | | |
| *HDAC1** | Class I | Controls embryonic stem cell differentiation (but not HDAC2) (9). Modulation of cell growth and apoptosis by down-regulation of p53. Also part of NuRD/Mi-2 ATP-dependent chromatin remodelling complex. |
| *HDAC2** | | Relevant role in haematopoiesis. Also part of NuRD/Mi-2 ATP-dependent chromatin remodelling complex. |
| *HDAC3* | | Modulation of cell growth and apoptosis by down-regulation of p53. |
| *HDAC8* | | |
| *HDAC4* | Class Iia | |
| *HDAC9* | | Protects neurons from apoptosis. |
| *HDAC5* | | |
| *HDAC7* | | |
| *HDAC6* | Class Iib | |
| *HDAC10* | | |
| *SIRT1** | Class III, NAD-dependent | Interacts with PRC2, non-histone deacetylase activity. Involved in normal ageing through resistance to cellular stress. Deacetylates p53. Located in nucleus and cytoplasm (10). |
| *SIRT2* | | Deacetylates alpha-tubulin. Located in the cytoplasm (10). |
| *SIRT3* | | Located in the mitochondria (10). |
| *SIRT4* | | |

| | | |
|---|---|---|
| *SIRT5* | | |
| *SIRT6* | | Located in the nucleus (10). H3K9 and H3K56 deacetylase activity. |
| *SIRT7* | | Located in the nucleus (10). |
| *HDAC11* | Class IV | |
| *ARID4A* | | Bridging molecule to recruit HDACs. |
| *TBL1XR1* | | Associates with HDAC3 (11). |
| *NCOR1* | | Forms complex with HDAC1. |
| *TRIM28 (KAP1)** | | Proposed to be a transcriptional repressor. Mediates apoptosis. through degradation of p53 (12). |
| Histone acetyltransferases | | |
| *EP300* | Type A, CBP/P300 family | Acetylates all four core histones, and non-histone proteins like p53 and MyoD (13). |
| *CREBBP (CBP)* | | Critical role in embryonic development, acetylates both histone and non-histone proteins. |
| *NCOA3* | | HAT activity not studied in detail. |
| *BRPF1 (TAF250)* | Type A | |
| *ATF2* | | Specifically acetylates H2B and H4 *in vitro*. |
| *KAT6A (MOZ)* | Type A, MYST family | Component of the MOZ/MORF complex, which has a histone H3 acetyltransferase activity. |
| *KAT6B (MORF)* | | |
| *KAT5 (TIP60)* | | |
| *KAT8 (MOF)* | | |
| *KAT7 (HBO1)* | | Responsible for the bulk of histone H4 acetylation *in vivo*. |
| *KAT2A (GCN5)* | Type A, GNAT family | |
| *KAT2B (PCAF)* | | |
| *HAT1* | Type B | |
| *ING4* | | Facilitates targeting of HBO1-mediated acetylation to H3K4me3 sites (14). |
| *SET* | HAT inhibitor | Promotes apoptosis. Inhibits p300/CBP and PCAF-mediated acetyltransferase. |
| Histone methyltransferases | | |
| *ASH1L (ASH1)* | | H3K36 HMT. |
| *ASH2L* | | H3K4 HMT. Complex with MLL |
| *ATF7IP (MCAF)** | | Required to stimulate SETDB1 activity, couples H3K9me3 with DNA methylation. |
| *DOT1L (KMT4)* | | H3K79 HMT. |
| *EHMT2 (G9a)* | | H3K9me1/me2, H3K27me HMT. |
| *EHMT1* | | H3K9me1/me2 HMT. |
| *EZH2** | | H3K27me1/me2/me3 HMT. Major role in stem cell identity maintenance. Also methylates GATA4. Catalytic subunit of PRC2 complex. |
| *MEN1* | | H3K4 HMT. Essential component of a MLL/SET1 HMT complex. Represses telomerase expression. Role in TGFB1-mediated inhibition of cell-proliferation. |
| *MLL* | | H3K4 HMT. Key regulator of development and haematopoiesis. |
| *MLL2* | | H3K4 HMT. |
| *MLL3* | | |
| *MLL4* | | H3K4 HMT. Required to control the bulk of H3K4me3 during oocyte growth and preimplantation. |
| *MLL5* | | H3K4me1/me2 HMT. Key regulator of haematopoiesis. |
| *NSD1 (KMT3B)* | | H3K36, H4K20 HMT. May influence transcription positively or negatively. |
| *PRDM2 (RIZ1)* | | H3K9 HMT. |
| *PRDM9* | | H3K4me3 HMT. Essential for meiotic progression. |
| *RBBP5* | | Complex with MLL. |
| *RTF1* | | Required for H3K4me3 HMT on stem cell pluripotency genes. |
| *SETD1A (SET1A)* | | H3K4 HMT. |
| *SETD1B (SET1B)* | | H3K4 HMT. |
| *SETD2 (KMT3A)* | | H3K36 HMT. |
| *SETD7 (SET7)* | | H3K4 HMT. |
| *SETD8 (KMT5A)* | | Trimethylates H4K20 (15). |
| *SETDB1 (ESET)* | | H3K9 HMT. |
| *SETDB2* | | H3K9 HMT. |
| *SMYD1* | | H3K4 HMT (16). |
| *SMYD2 (KMT3C)* | | H3K4me, H3K36me2 HMT. Also methylates TP53 and RB1. |
| *SMYD3* | | H3K4me2/me3 HMT. |
| *SUV39H1 (KMT1A)* | | H3K9me3 HMT, uses H3K9me1 as substrate. |
| *SUV39H2 (KMT1B)* | | |
| *SUV420H1 (KMT5B)* | | H4K20me3 HMT. Key in constitutive heterochromatin formation at pericentormeric regions. |
| *SUV420H2 (KMT5C)* | | |
| *TRIM28 (KAP1)** | | Mediates silencing by recruiting SET1 H3K9me3 HMT and HDAC NuRD complex. Mediates apoptosis through degradation of p53 (12). |

| | | |
|---|---|---|
| *WDR5* | | Complex with MLL. |
| Histone demethylases | | |
| *KDM1A (LSD1)** | | H3K4me2/me1, H3K9 HDM, also demethylates and stabilizes DNMT1. |
| *KDM1B (LSD2)** | | H3K4me2/me1 HDM. Required for *de novo* DNA methylation of a subset of imprinted genes during oogenesis. |
| *KDM2A* | | H3K36me2 HDM. Required to maintain heterochromatic state at centromeres. |
| *KDM2B* | | H3K4me3, H3K36me2 HDM. Represses rRNA genes. |
| *KDM3A* | | H3K9me2/me1 HDM. |
| *KDM3B* | | H3K9 HDM. |
| *KDM4A* | | H3K9me3, H3K36me3 HDM. |
| *KDM4B* | | H3K9me3 HDM. |
| *KDM4C* | | H3K9me3, H3K36me3 HDM. |
| *KDM4D* | | H3K9me3/me2 HDM. |
| *KDM5A (RBP2)* | | H3K4me2/me3 HDM. Prominent role in cell differentiation and senescence (17). |
| *KDM5B (PLU1)* | | H3K4me3/me2/me1 HDM. |
| *KDM5C (SMCX)* | | H3K4me3/me2 HDM. Participates in the repression of neuronal genes. |
| *KDM5D (SMCY)* | | H3K4me3/me2 HDM. |
| *KDM6A (UTX)* | | H3K27me2/me3 HDM. Regulation of HOX gene expression. |
| *KDM6B (JMJD3)* | | |
| *JHDM1D (KDM7A)* | | H3K9me2, H3K27me2, H4K20me1 HDM. Required for brain development. |
| *KDM8 (JMJD5)* | | H3K36me2 HDM. Required for G2/M cell cycle progression. |
| *JMJD1C (TRIP8)* | | H3K9 HDM. |
| *JMJD6* | | H3R2, H4R3 HDM. Key regulator of haematopoietic differentiation. |
| *PHF2* | | H3K9me2 HDM. |
| *PHF8* | | H3K9me1/me2, H3K27me2, H4K20me1 HDM. Key role in cell cycle progression. |
| *UTY* | | H3K27me3/me2/me1 HDM (18). |
| *JARID2 (JMJ)** | | Essential role in embryonic development, inhibits PRC2 trimethylation of H3K27 (19). |
| DNA methyltransferases | | |
| *DNMT1* | | Maintainins methylation patterns established in development. |
| *DNMT3A* | | Genome-wide *de novo* methylation, essential for the establishment of DNA methylation patterns during development. |
| *DNMT3B* | | |
| *DNMT3L* | | Catalytically inactive, but essential for DNMT3A and DNMT3B function. |
| *MECP2* | | Essential for embryonic development. Specifically bind methylated DNA and repress transcription at methylated promoters. |
| *MBD1* | | |
| *MBD2** | | |
| *MBD4* | | |
| *ATF7IP (MCAF)** | | Mediates MBD1 transcriptional repression, couples H3K9me3 with DNA methylation. |
| *KDM1A (LSD1)** | | HDM, also demethylates and stabilizes DNMT1. |
| *KDM1B (LSD2)** | | HDM, required for *de novo* DNA methylation of a subset of imprinted genes during oogenesis. |
| DNA demethylases | | |
| *TET1* | Converts 5mC to 5hmC | Putative role in DNA demethylation (20). |
| *TET2* | | |
| *AICDA (AID)* | | May play a role in DNA demethylation. |
| *TDG* | | Essential for DNA demethylation (21). |
| ATP-dependent chromatin remodelling | | |
| *SMARCA2 (BRM)* | SWI/SNF complex is required for transcriptional activation of genes normally repressed by chromatin (22). | Catalytic component of SWI/SNIF complex (23). |
| *SMARCA4 (BRG1)* | | Essential for the maintenance of multipotent neural stem cells. |
| *SMARCB1 (BAF47)* | | |
| *SMARCC1* | | |
| *SMARCC2* | | |
| *SMARCD1* | | |
| *SMARCD2* | | |
| *SMARCD3* | | |
| *SMARCE1 (BAF57)* | | |
| *ARID1A* | | |
| *ARID1B (BAF250B)* | | |
| *ARID2 (BAF200)* | | Required for the stability of the SWI/SNF chromatin remodelling complex SWI/SNF-B. |
| *ACTL6A (BAF53A)* | | Required for maximal SMARCA4 activity and for the association of the SWI/SNF complex with chromatin. |
| *ACTL6B (BAF53B)* | | |
| *DPF1 (BAF45B)* | | |
| *DPF2 (BAF45D)* | | |

| | | |
|---|---|---|
| DPF3 (BAF45C) | | |
| EP400 | | Regulates nucleosome stability during DNA repair (24). |
| PBRM1 | | Regulator of cell proliferation. |
| PHF10 (BAF45A) | | Required for the proliferation of neural progenitors. |
| MTA1 | | |
| MTA2 | | |
| MTA3 | | Maintenance of the normal epithelial architecture through the repression of SNAI1 transcription in a HDAC-dependent manner. |
| CHD3 (Mi-2α) | NuRD/Mi-2 complex has ATP-dependent chromatin remodelling activity and HDAC activity | |
| CHD4 (Mi-2β) | | Main component of the NuRD/Mi-2 complex. |
| GATAD2A | | |
| GATAD2B | | |
| HDAC1* | | |
| HDAC2* | | |
| MBD2* | | Essential for embryonic development. Also bind methylated DNA. |
| RBBP4 (RBAP46)* | | Also part of PRC2 complex. |
| RBBP7 (RBAP48)* | | |
| INO80 | INO80 complex has DNA- and nucleosome-activated ATPase activity and catalyzes ATP-dependent nucleosome sliding (25). | |
| TFPT | | Putative regulatory component of the INO80 complex |
| YY1* | | Also interacts with PRC2 and is required for EZH2-mediated H3K27me3 (7). |
| SMARCA1 (SNF2L) | | |
| SMARCA5 (SNF2H) | ISWI complex mobilizes mononucleosomes away from DNA ends without changing the arrangement of DNA on the surface of the histone octamer (22). | Required for replication of pericentric heterochromatin in S-phase specifically in conjunction with BAZ1A. |
| BAZ1A (ACF1) | | |
| BAZ1B (WSTF) | | Acts as a mark that distinguishes between apoptotic and repair responses to genotoxic stress. Maintenance of chromatin structures during DNA replication processes. |
| BAZ2A (TIP5) | | |
| BPTF | | Binds H3K4me3. |
| CHRAC1 | | |
| POLE3 | | |
| RSF1 | | |
| RBBP4 (RBAP46)* | | Also part of PRC2 complex. |
| RBBP7 (RBAP48)* | | |
| CHD1 | | Required for the maintenance of open chromatin and pluripotency in ESC. |
| CHD2 | | SNF2-related helicase/ATPase domains. |
| HNF1A | | Possible regulation of transcription through chromatin remodelling (26). |
| IKZF1* | | Targets NuRD/Mi-2 and SWI/SNF complexes in a single complex. |
| Global chromatin regulators | | |
| LMNA | lamin A/C | Global heterochromatic changes induced by lamin perturbation are often mirrored by altered levels of chromatin-associated epigenetic histone marks (27). |
| LMNB1 | lamin B1 | |
| LMNB2 | lamin B2 | |
| Other chromatin regulators | | |
| BAG6 | Complex EP300 | p300-mediated p53 acetylation upon DNA damage. May mediate H3K4me2. |
| ATRX | ATRX-DAXX complex | Thought to regulate deposition of H3.3 at heterochromatic regions of the genome, including telomeres (28). |
| DAXX | | |
| MUM1 | | Opens chromatin to facilitate DNA damage repair (29). |

*Genes with more than one function in chromatin remodelling appear more than once in the table.

(a) HGNC HUGO gene names. In parenthesis, common alternative gene names.

(b) Gene function provided by Uniprot, unless otherwise stated.(30)

**Table S2. Described oncogenic alterations in Chromatin Regulatory Factors.** This is an exhaustive compilation of alterations(*) reported in CRFs not included in Table 1. Gene names correspond to HUGO HGNC approved symbols. In bold typeface, genes included in the Cancer Gene Census (CGC) (31). ALL: Acute Lymphocytic Leukaemia; AML: Acute Myeloid Leukaemia; B-ALL: B Acute Lymphoblastic Leukaemia; B-NHL: B-cell non-Hodgkin Lymphoma; CLL: Chronic Lymphocytic Leukaemia; ccOC: Clear Cell Ovarian Carcinoma; ccRCC: clear-cell Renal Cell Carcinoma; CMML: Chronic Myelomonocytic leukaemia; ESCC: Oesophageal Squamous Cell Carcinoma; FL: Follicular Lymphoma; HCC: Hepatocellular Carcinoma; HL: Hodgkin Lymphoma; HNSCC: Head and Neck Squamous Cell Carcinoma; MCL: Mantle cell Lymphoma; MDS: Myelodysplastic Syndrome; MSI: Microsatellite instability; NMSC: Non-Melanoma Skin Cancer; NSCLC: Non-Small Cell Lung Carcinoma; OSCC: Oral Squamous Cell Carcinoma; RCC: Renal Cell Carcinoma; T-ALL: T Acute Lymphoblastic Leukaemia.

*Evidence based solely on cancer cell lines is excluded from this table. Only evidence in human samples have been used. Effects of pharmacological inhibition are not included. Germline polymorphisms are excluded.

| Gene | Literature evidence |
|---|---|
| *AEBP2* | Deleted in AML (32). |
| *ATF2* | Over-expressed in melanoma (33). |
| *BAZ1A* | Amplified in ESCC (34).<br>Deleted in papillary type 2 RCC (35). |
| *BMI1* | Over-expressed in B-NHL, leukaemia, MCL, medulloblastoma, neuroblastoma, NSCLC (36) and prostate tumours (37). |
| *CBX2* | Over-expressed in breast cancer (38). |
| *CBX3* | Over-expressed in osteosarcoma (39), myxoid liposarcoma, colon, breast, esophageal, cervical, and lung tumours (40). |
| *CBX7* | Over-expressed in lymphoma (41).<br>Down-regulated in bladder (42), and aggressive gastric (43), pancreatic (44) and thyroid cancer (45). |
| *CHD1* | Mutated in high MSI gastric and colorectal cancers (46).<br>Deleted in prostate cancer (47). |
| ***CREBBP*** | Mutated in AML, ALL, DLBCL, N-NHL (CGC), bladder (48), medulloblastoma (49) and SCLC (50). LOH in lung (51). |
| ***DAXX*** | Mutated in paediatric glioblastoma and neuroendocrine pancreatic tumours (CGC).<br>Over-expressed in prostate cancer (52). |
| *DNMT1* | Over-expressed in AML (53), gliomas (54) and pancreatic tumours (55). |
| *DNMT3B* | Over-expressed in breast (56), colorectal and stomach (57), prostate cancer (58), advanced stages of DLBCL (59). |
| *DNMT3L* | Over-expressed in testicular embryonal carcinoma (60).<br>Loss of methylation and consequent over-expression in cervical cancer (61). |
| *EHMT2* | Over-expressed in bladder (62), resistant cervical (63) and aggressive lung tumours (64). |
| *EPC1* | Mutated in pancreatic cancer (65). |
| *EZH1* | Over-expressed and amplified in myeloproliferative neoplasms (66). |
| ***EZH2*** | Mutated in DLBCL (CGC), MDS (67).<br>Over-expressed in bladder, breast, colon, liver, melanoma and prostate tumours; DLBCL, HL and MCL (36). |
| *GATAD2B* | Deleted in OSCC (68). |
| *HDAC1* | Over-expressed in HCC (69).<br>Down-regulated in aggressive breast tumours (70). |
| *HDAC2* | Mutated in colon cancer with microsatellite instability (71).<br>Over-expressed in gastrointestinal tumours (72), prostate (73), aggressive HCC (74), lung (75), cervical (76), ovarian and endometrial endometrioid carcinomas (77). |

| | |
|---|---|
| *HDAC3* | Over-expressed in gastrointestinal tumours (72), b-cell lymphomas (78) and CLL (79). |
| *HDAC4* | Mutated in melanoma (80) and breast cancer (81).<br>Over-expressed in T-ALL (82) and treatment-resistant ovarian tumours (83). |
| *HDAC5* | Over-expressed in B-ALL (82) and aggressive medulloblastoma (84). |
| *HDAC6* | Over-expressed in HCC (85), cisplatin-resistant NSCLC (86) and breast tumours with good prognosis (87).<br>Down-regulated in CLL (79). |
| *HDAC7* | Over-expressed in pancreatic adenocarcinoma (88) and aggressive childhood ALL (82). |
| *HDAC8* | Over-expressed in aggressive neuroblastoma (89). |
| *HDAC9* | Over-expressed in high grade medulloblastoma (84) and childhood ALL with poor prognosis (82).<br>Amplified in OSCC (68). |
| *HDAC10* | Down-regulated in adrenocortical tumours (90), CLL (91) and aggressive NSCLC (92). |
| *HNF1A* | Mutated in neuroendocrine tumours (93), endometrial cancer (94), high MSI CRC (95) and hepatocellular adenoma (96).<br>Down-regulated in aggressive HCC (97). |
| ***IKZF1*** | Mutated in ALL, DLBCL (CGC).<br>Deleted in aggressive paediatric B-ALL (98). |
| *ING4* | Down-regulated in HNSCC (99), melanoma (100), gastric adenocarcinoma (101), lung tumours (102) and colorectal cancer (103).<br>Deleted in HNSCC (99) and breast tumours (104). |
| *JARID2* | Mutated in NSCLC (105).<br>Deleted in AML (32). |
| *JMJD1C* | Over-expressed in pancreatic ductal adenocarcinoma (106). |
| *JMJD6* | Over-expressed in aggressive breast tumours (107). |
| *KAT5* | Down-regulated in gastric cancer (108), aggressive melanoma (109) and advanced colorectal carcinoma (110). |
| *KAT6A* | Translocated in AML (111). |
| *KAT6B* | Translocated in AML (111) and benign uterine tumours (112). |
| *KAT7* | Over-expressed in testicular, breast, ovarian, bladder, oral and oesophageal carcinomas (113). |
| *KAT8* | Down-regulated in breast carcinoma and medulloblastoma (114). |
| *KDM1A* | Over-expressed in NSCLC (115), highly malignant sarcomas (116), bladder (117) and aggressive prostate tumours (118).<br>Down-regulated in breast carcinoma (119). |
| *KDM2A* | Down-regulated in prostate cancer (120). |
| *KDM2B* | Over-expressed in ALL, AML (121) and pancreatic ductal adenocarcinoma (106). |
| *KDM3A* | Over-expressed in prostate cancer (122) and RCC (123). |
| *KDM3B* | Over-expressed in ALL (124) and prostate cancer (122). |
| *KDM4A* | Over-expressed in breast (125) and prostate cancer (122).<br>Down-regulated in bladder tumours (126). |
| *KDM4B* | Over-expressed in gastric cancer (127). |
| *KDM4C* | Over-expressed and amplified in breast cancer (128). |
| ***KDM5A*** | Mutated in AML (CGC).<br>Down-regulated in melanoma (129).<br>Over-expressed in breast tumours with good prognosis (130) and in pancreatic ductal adenocarcinoma (106). |
| *KDM5B* | Over-expressed in breast tumours, prostate cancer (122) and uveal melanoma (131). |
| *KDM6B* | Over-expressed in HL (132) and pancreatic ductal adenocarcinoma (106). |
| *LMNA* | Over-expressed in aggressive colorectal cancer (133).<br>Down-regulated in DLBCL (134), ALL and NHL (135). |
| *LMNB1* | Over-expressed in HCC (136) and colorectal tumours (137). |
| *MBD4* | Mutated in sporadic colon cancer (138) and HNPCC with MSI (139). |

| | |
|---|---|
| *MECP2* | Over-expressed in breast tumours (140). |
| ***MEN1*** | Mutated in pancreas, parathyroid (CGC) and in lung carcinoids (141).<br>MLL-fusion partner in leukaemias (142). |
| *MLL5* | Down-regulated in poor prognosis AML (143). |
| *MTA1* | Over-expressed in OSCC, ESCC, early NSCLC, HCC, osteosarcoma, and colorectal, pancreatic, endometrial, ovarian, prostate, breast and gastric cancers. It is one of the most commonly over-expressed genes in human tumours (144). |
| *MTA2* | Over-expressed in NSCLC (145), aggressive HCC (146) and epithelial ovarian cancer (147). |
| *MUM1* | Over-expressed in aggressive PCLBCL (148) and CLL (149), DLBCL and HL (150). |
| *NCOA3* | Over-expressed in HCC, breast (151), urothelial carcinoma of the bladder (152), NSCLC (153) and prostate tumours (154).<br>Amplified in breast cancer (155).<br>Fusion partner of KAT6A in AML (156). |
| *PCGF2* | Over-expressed in aggressive medulloblastoma (157).<br>Down-regulated in breast tumours (158) and high-grade prostate cancer (159). |
| *PHC1* | Over-expressed in ALL (36). |
| *PHF8* | Over-expressed in prostate cancer (122). |
| *PHF19* | Over-expressed in colon, skin, lung, rectal, cervical, uterine and hepatic tumours (36). |
| *PRDM2* | Mutated in endometrial, gastrointestinal (160) and colon tumours with MSI (161), melanoma (162).<br>Over-expressed in ALL (163).<br>Down-regulated in ESCC (164), neuroblastoma (165), HCC (166), epithelial ovarian carcinoma (167), thyroid carcinoma (168) and AML (163).<br>Deleted in parathyroid tumours (169). |
| *RBBP4* | Over-expressed in HPV-positive oropharyngeal tumours (170).<br>Down-regulated in mucoepidermoid carcinoma (171). |
| *RBBP5* | Amplified in glioblastomas (172). |
| *RBBP7* | Over-expressed in NSCLC (173) and breast tumours (174). |
| *RING1* | Over-expressed in prostate tumours (37). |
| *RSF1* | Over-expressed in NSCLC (175), urinary bladder (176), colon (177), gallbladder (178), nasopharyngeal (179) and ovarian aggressive carcinomas (180).<br>Amplified in aggressive ovarian carcinoma (181). |
| ***SET*** | Mutated in AML (CGC).<br>Over-expressed in colorectal adenocarcinoma (182) and paediatric B-ALL and T-ALL (183). |
| *SET8* | Over-expressed in aggressive breast tumours (184). |
| *SETDB2* | Deleted in CLL (185). |
| *SIRT1* | Over-expressed in leukaemia, prostate, skin and colon cancers (186)<br>Down-regulated in breast tumours and HCC (187). |
| *SIRT2* | Down-regulated in gliomas (188). |
| *SIRT3* | Down-regulated in HCC (189). |
| *SIRT6* | Down-regulated in pancreas and colorectal cancer (190).<br>Deleted in colorectal cancer (190). |
| *SIRT7* | Over-expressed in breast (191) and thyroid carcinoma (192). |
| ***SMARCB1*** | Mutated in malignant rhabdoid tumours (CGC). |
| *SMARCC1* | Over-expressed in prostate cancer (193) and precancerous cervical lesions (194).<br>High expression correlates with good prognosis in colorectal cancer (195). |
| *SMARCD1* | Mutated in breast tumours (196). |
| *SMARCD3* | Over-expressed in advanced neuroblastoma (197). |
| *SMARCE1* | Over-expressed in aggressive endometrial carcinoma (198). |
| *SMYD2* | Over-expressed in ESCC (199). |
| *SMYD3* | Over-expressed in colorectal cancer (200). |
| ***SUZ12*** | Mutated in endometrial stromal tumours (CGC).<br>Over-expressed in breast, colon, liver (36) and ovarian tumours (201). |

| | |
|---|---|
| | Amplified in MCL (202). |
| *TBL1XR1* | Over-expressed in SCC (203).<br>Deleted in ALL (204) and PCNSL (205). |
| *TET1* | Mutated in T-ALL (206).<br>Down-regulated in prostate and breast tumours (207). |
| ***TFPT*** | Mutated in pre-B ALL (CGC). |
| *TRIM28* | Over-expressed in colorectal tumours (208), gastric cancer cell lines (209), NSCLC and breast (210).<br>Over-expression predicts better survival in early lung tumours (210).<br>High expression indicates good prognosis in gastric cancer (209). |
| *YY1* | Over-expressed in prostate, colon, ovary, breast, bone, liver, lung, bladder, cervix, skin and blood (DLBCL, AML, CML, ALL, HL, BL, MCL, CLL and FL) cancers (211).<br>Down-regulated in melanomas, paediatric osteosarcomas and urothelial carcinomas (211).<br>There are contradictory results on the prognostic significance of YY1 in cancer (211). |

**Table S3. Mutually exclusivity test for mutations in genes coding proteins that act in the same complex.**

| Protein complex | Site | Gene 1 | Gene 2 | *P* value Fisher Test |
|---|---|---|---|---|
| SWISNF | Bladder | ARID2 | ARID1A | 0.02 |
| ISWI | Bladder | BAZ2A | BPTF | 0.03 |
| NURDMI2 | Bladder | CHD3 | CHD4 | $P < 10^{-16}$ |
| SWISNF | Breast | ARID2 | ARID1A | 0.01 |
| SWISNF | Breast | SMARCA2 | ARID1A | 0.01 |
| SWISNF | Breast | SMARCA4 | ARID1A | $P < 10^{-16}$ |
| ISWI | Breast | BAZ2A | BPTF | $1.08*10^{-5}$ |
| NURDMI2 | Breast | CHD3 | CHD4 | $1.13*10^{-6}$ |
| PRC1 | Breast | BAP1 | PHC3 | $P < 10^{-16}$ |
| SWISNF | Head & Neck | ARID2 | SMARCA4 | 0.05 |
| NURDMI2 | Head & Neck | CHD3 | CHD4 | $1.98*10^{-5}$ |
| PRC1 | Head & Neck | BAP1 | PHC3 | 0.01 |
| SWISNF | Lung | SMARCA2 | SMARCA4 | 0.04 |
| SWISNF | Lung | SMARCA2 | ARID1A | 0.04 |
| ISWI | Lung | BAZ2A | BPTF | $P < 10^{-16}$ |
| NURDMI2 | Lung | CHD3 | CHD4 | $8.35*10^{-9}$ |
| PRC1 | Lung | BAP1 | PHC3 | 0 |
| SWISNF | Ovary | ARID2 | SMARCA4 | 0.05 |
| SWISNF | Uteri | PBRM1 | ARID1A | 0.02 |
| SWISNF | Uteri | SMARCA4 | ARID1A | 0 |
| ISWI | Uteri | BAZ2A | BPTF | 0 |
| NURDMI2 | Uteri | CHD3 | CHD4 | $5.29*10^{-8}$ |

**Table S4. Gene regulatory modules collected for the analysis.**

| Group | Name | Cell type | Nº of genes | Source |
|---|---|---|---|---|
| EP300 | EP300 ES | ES | 1191 | Lister *et al*. 2009 (212) |
| | EP300 CD4 | CD4 | 3792 | Wang *et al*. 2009 (213) |
| Activating histone marks | H3K4me3 ES | ES | 12312 | ENCODE (214) |
| | H3K4me3 CD4 | CD4 | 11423 | Barski *et al*. 2007 (215) |
| | H3K4me3 gm12878 | gm12878 | 11771 | ENCODE (214) |
| | H3K9ac ES | ES | 10489 | ENCODE (214) |
| | H3K9ac CD4 | CD4 | 6906 | Wang *et al*. 2009 (213) |
| | H3K9ac gm12878 | gm12878 | 9918 | ENCODE (214) |
| Repressive histone marks | H3K27me3 ES | ES | 6665 | ENCODE (214) |
| | H3K27me3 CD4 | CD4 | 5207 | Wang *et al*. 2009 (213) |
| | H3K27me3 gm12878 | gm12878 | 6099 | ENCODE (214) |
| Replication Timing | Late RT ES | ES | 918 | Hansen *et al*. 2010 (216) |
| | Late RT lymphoid | lymphoid | 260 | Hansen *et al*. 2010 (216) |

## *Supplementary references*


1. Cao R, Zhang Y. SUZ12 Is Required for Both the Histone Methyltransferase Activity and the Silencing Function of the EED-EZH2 Complex. Molecular Cell. 2004 Jul 2;15(1):57–67.

2. Kuzmichev A, Nishioka K, Erdjument-Bromage H, Tempst P, Reinberg D. Histone methyltransferase activity associated with a human multiprotein complex containing the Enhancer of Zeste protein. Genes Dev. 2002 Nov 15;16(22):2893–905.

3. Cai L, Rothbart SB, Lu R, Xu B, Chen W-Y, Tripathy A, et al. An H3K36 Methylation-Engaging Tudor Motif of Polycomb-like Proteins Mediates PRC2 Complex Targeting. Mol Cell. 2012 Dec 22;

4. Ballaré C, Lange M, Lapinaite A, Martin GM, Morey L, Pascual G, et al. Phf19 links methylated Lys36 of histone H3 to regulation of Polycomb activity. Nat Struct Mol Biol. 2012 Dec;19(12):1257–65.

5. Abdel-Wahab O, Adli M, LaFave LM, Gao J, Hricik T, Shih AH, et al. ASXL1 mutations promote myeloid transformation through loss of PRC2-mediated gene repression. Cancer Cell. 2012 Aug 14;22(2):180–93.

6. Li X, Isono K, Yamada D, Endo TA, Endoh M, Shinga J, et al. Mammalian Polycomb-Like Pcl2/Mtf2 Is a Novel Regulatory Component of PRC2 That Can Differentially Modulate Polycomb Activity both at the Hox Gene Cluster and at Cdkn2a Genes. Mol Cell Biol. 2011 Jan;31(2):351–64.

7. Caretti G, Di Padova M, Micales B, Lyons GE, Sartorelli V. The Polycomb Ezh2 methyltransferase regulates muscle gene expression and skeletal muscle differentiation. Genes Dev. 2004 Nov 1;18(21):2627–38.

8. Trojer P, Cao AR, Gao Z, Li Y, Zhang J, Xu X, et al. L3MBTL2 protein acts in concert with PcG protein mediated monoubiquitination of H2A to establish a repressive chromatin structure. Mol Cell. 2011 May 20;42(4):438–50.

9. Dovey OM, Foster CT, Cowley SM. Histone deacetylase 1 (HDAC1), but not HDAC2, controls embryonic stem cell differentiation. PNAS. 2010 May 4;107(18):8242–7.

10. Rajendran R, Garva R, Krstic-Demonacos M, Demonacos C. Sirtuins: Molecular Traffic Lights in the Crossroad of Oxidative Stress, Chromatin Remodeling, and Transcription. J Biomed Biotechnol [Internet]. 2011 [cited 2013 Jan 21];2011. Available from: http://www.ncbi.nlm.nih.gov/pmc/articles/PMC3168296/

11. Yoon H-G, Chan DW, Huang Z-Q, Li J, Fondell JD, Qin J, et al. Purification and functional characterization of the human N-CoR complex: the roles of HDAC3, TBL1 and TBLR1. EMBO J. 2003 Mar 17;22(6):1336–46.

12. Iyengar S, Farnham PJ. KAP1 Protein: An Enigmatic Master Regulator of the Genome. J Biol Chem. 2011 Jul 29;286(30):26267–76.

13. Hodawadekar SC, Marmorstein R. Chemistry of acetyl transfer by histone modifying enzymes: structure, mechanism and implications for effector design. Oncogene. 2007;26(37):5528–40.

14. Palacios A, Moreno A, Oliveira BL, Rivera T, Prieto J, García P, et al. The dimeric structure and the bivalent recognition of H3K4me3 by the tumor suppressor ING4 suggests a mechanism for enhanced targeting of the HBO1 complex to chromatin. J Mol Biol. 2010 Mar 5;396(4):1117–27.

15. Nishioka K, Rice JC, Sarma K, Erdjument-Bromage H, Werner J, Wang Y, et al. PR-Set7 is a nucleosome-specific methyltransferase that modifies lysine 20 of histone H4 and is associated with silent chromatin. Mol Cell. 2002 Jun;9(6):1201–13.

16. Xu S, Zhong C, Zhang T, Ding J. Structure of human lysine methyltransferase Smyd2 reveals insights into the substrate divergence in Smyd proteins. J Mol Cell Biol. 2011 Oct 1;3(5):293–300.

17. Benevolenskaya EV, Murray HL, Branton P, Young RA, Kaelin Jr. WG. Binding of pRB to the PHD Protein RBP2 Promotes Cellular Differentiation. Molecular Cell. 2005 Jun 10;18(6):623–35.

18. Islam ABMMK, Richter WF, Jacobs LA, Lopez-Bigas N, Benevolenskaya EV. Co-Regulation of Histone-Modifying Enzymes in Cancer. PLoS One [Internet]. 2011 Aug 23 [cited 2013 Jan 21];6(8). Available from: http://www.ncbi.nlm.nih.gov/pmc/articles/PMC3160334/

19. Peng JC, Valouev A, Swigut T, Zhang J, Zhao Y, Sidow A, et al. Jarid2/Jumonji Coordinates Control of PRC2 Enzymatic Activity and Target Gene Occupancy in Pluripotent Cells. Cell. 2009 Dec 24;139(7):1290–302.

20. Tahiliani M, Koh KP, Shen Y, Pastor WA, Bandukwala H, Brudno Y, et al. Conversion of 5-methylcytosine to 5-hydroxymethylcytosine in mammalian DNA by MLL partner TET1. Science. 2009 May 15;324(5929):930–5.

21. Cortellino S, Xu J, Sannai M, Moore R, Caretti E, Cigliano A, et al. Thymine DNA Glycosylase Is Essential for Active DNA Demethylation by Linked Deamination-Base Excision Repair. Cell. 2011 Jul 8;146(1):67–79.

22. Kassabov SR, Zhang B, Persinger J, Bartholomew B. SWI/SNF Unwraps, Slides, and Rewraps the Nucleosome. Molecular Cell. 2003 Feb;11(2):391–403.

23. Harikrishnan KN, Chow MZ, Baker EK, Pal S, Bassal S, Brasacchio D, et al. Brahma links the SWI/SNF chromatin-remodeling complex with MeCP2-dependent transcriptional silencing. Nature Genetics. 2005;37(3):254–64.

24. Xu Y, Sun Y, Jiang X, Ayrapetov MK, Moskwa P, Yang S, et al. The p400 ATPase regulates nucleosome stability and chromatin ubiquitination during DNA repair. J Cell Biol. 2010 Oct 4;191(1):31–43.

25. Jin J, Cai Y, Yao T, Gottschalk AJ, Florens L, Swanson SK, et al. A Mammalian Chromatin Remodeling Complex with Similarities to the Yeast INO80 Complex. J Biol Chem. 2005 Dec 16;280(50):41207–12.

26. Pontoglio M, Faust DM, Doyen A, Yaniv M, Weiss MC. Hepatocyte nuclear factor 1alpha gene inactivation impairs chromatin remodeling and demethylation of the phenylalanine hydroxylase gene. Mol Cell Biol. 1997 Sep;17(9):4948–56.

27. Dittmer TA, Misteli T. The lamin protein family. Genome Biol. 2011;12(5):222.

28. Elsässer SJ, Allis CD, Lewis PW. New Epigenetic Drivers of Cancers. Science. 2011 Mar 4;331(6021):1145–6.

29. Huen MSY, Huang J, Leung JWC, Sy SM-H, Leung KM, Ching Y-P, et al. Regulation of chromatin architecture by the PWWP domain-containing DNA damage-responsive factor EXPAND1/MUM1. Mol Cell. 2010 Mar 26;37(6):854–64.

30. Ley TJ, Ding L, Walter MJ, McLellan MD, Lamprecht T, Larson DE, et al. DNMT3A Mutations in Acute Myeloid Leukemia. New England Journal of Medicine. 2010;363(25):2424–33.

31. Futreal PA, Coin L, Marshall M, Down T, Hubbard T, Wooster R, et al. A census of human cancer genes. Nat Rev Cancer. 2004 Mar;4(3):177–83.

32. Puda A, Milosevic JD, Berg T, Klampfl T, Harutyunyan AS, Gisslinger B, et al. Frequent deletions of JARID2 in leukemic transformation of chronic myeloid malignancies. Am J Hematol. 2012 Mar;87(3):245–50.

33. Bhoumik A, Ronai Z. ATF2: a transcription factor that elicits oncogenic or tumor suppressor activities. Cell Cycle. 2008 Aug;7(15):2341–5.

34. Yasui K, Imoto I, Fukuda Y, Pimkhaokham A, Yang ZQ, Naruto T, et al. Identification of target genes within an amplicon at 14q12-q13 in esophageal squamous cell carcinoma. Genes Chromosomes Cancer.

2001 Oct;32(2):112–8.

35. Krill-Burger JM, Lyons MA, Kelly LA, Sciulli CM, Petrosko P, Chandran UR, et al. Renal cell neoplasms contain shared tumor type-specific copy number variations. Am J Pathol. 2012 Jun;180(6):2427–39.

36. Sparmann A, Lohuizen M van. Polycomb silencers control cell fate, development and cancer. Nature Reviews Cancer. 2006 Nov 1;6(11):846–56.

37. Van Leenders GJLH, Dukers D, Hessels D, van den Kieboom SWM, Hulsbergen CA, Witjes JA, et al. Polycomb-group oncogenes EZH2, BMI1, and RING1 are overexpressed in prostate cancer with adverse pathologic and clinical features. Eur Urol. 2007 Aug;52(2):455–63.

38. Parris TZ, Danielsson A, Nemes S, Kovács A, Delle U, Fallenius G, et al. Clinical Implications of Gene Dosage and Gene Expression Patterns in Diploid Breast Carcinoma. Clin Cancer Res. 2010 Aug 1;16(15):3860–74.

39. Saini V, Hose CD, Monks A, Nagashima K, Han B, Newton DL, et al. Identification of CBX3 and ABCA5 as Putative Biomarkers for Tumor Stem Cells in Osteosarcoma. PLoS One [Internet]. 2012 Aug 3 [cited 2013 Jan 21];7(8). Available from: http://www.ncbi.nlm.nih.gov/pmc/articles/PMC3411700/

40. Takanashi M, Oikawa K, Fujita K, Kudo M, Kinoshita M, Kuroda M. Heterochromatin Protein 1γ Epigenetically Regulates Cell Differentiation and Exhibits Potential as a Therapeutic Target for Various Types of Cancers. Am J Pathol. 2009 Jan;174(1):309–16.

41. Scott CL, Gil J, Hernando E, Teruya-Feldstein J, Narita M, Martínez D, et al. Role of the chromobox protein CBX7 in lymphomagenesis. Proc Natl Acad Sci U S A. 2007 Mar 27;104(13):5389–94.

42. Hinz S, Kempkensteffen C, Christoph F, Krause H, Schrader M, Schostak M, et al. Expression parameters of the polycomb group proteins BMI1, SUZ12, RING1 and CBX7 in urothelial carcinoma of the bladder and their prognostic relevance. Tumour Biol. 2008;29(5):323–9.

43. Zhang X-W, Zhang L, Qin W, Yao X-H, Zheng L-Z, Liu X, et al. Oncogenic role of the chromobox protein CBX7 in gastric cancer. J Exp Clin Cancer Res. 2010 Aug 19;29(1):114.

44. Karamitopoulou E, Pallante P, Zlobec I, Tornillo L, Carafa V, Schaffner T, et al. Loss of the CBX7 protein expression correlates with a more aggressive phenotype in pancreatic cancer. Eur J Cancer. 2010 May;46(8):1438–44.

45. Pallante P, Federico A, Berlingieri MT, Bianco M, Ferraro A, Forzati F, et al. Loss of the CBX7 Gene Expression Correlates with a Highly Malignant Phenotype in Thyroid Cancer. Cancer Res. 2008 Aug 15;68(16):6770–8.

46. Kim MS, Chung NG, Kang MR, Yoo NJ, Lee SH. Genetic and expressional alterations of CHD genes in gastric and colorectal cancers. Histopathology. 2011 Apr;58(5):660–8.

47. Liu W, Lindberg J, Sui G, Luo J, Egevad L, Li T, et al. Identification of novel CHD1-associated collaborative alterations of genomic structure and functional assessment of CHD1 in prostate cancer. Oncogene. 2012 Aug 30;31(35):3939–48.

48. Gui Y, Guo G, Huang Y, Hu X, Tang A, Gao S, et al. Frequent mutations of chromatin remodeling genes in transitional cell carcinoma of the bladder. Nature Genetics. 2011;43(9):875–8.

49. Robinson G, Parker M, Kranenburg TA, Lu C, Chen X, Ding L, et al. Novel mutations target distinct subgroups of medulloblastoma. Nature. 2012 Aug 2;488(7409):43–8.

50. Peifer M, Fernández-Cuesta L, Sos ML, George J, Seidel D, Kasper LH, et al. Integrative genome analyses identify key somatic driver mutations of small-cell lung cancer. Nature Genetics. 2012;44(10):1104–10.

51. Dokmanovic M, Clarke C, Marks PA. Histone Deacetylase Inhibitors: Overview and Perspectives. Mol Cancer Res. 2007 Oct 1;5(10):981–9.

52. Kwan P-S, Lau CC, Chiu YT, Man C, Liu J, Tang K, et al. Daxx regulates mitotic progression and prostate cancer predisposition. Carcinogenesis. 2012 Dec 13;

53. Mizuno S, Chijiwa T, Okamura T, Akashi K, Fukumaki Y, Niho Y, et al. Expression of DNA methyltransferases DNMT1,3A, and 3B in normal hematopoiesis and in acute and chronic myelogenous leukemia. Blood. 2001 Mar 1;97(5):1172–9.

54. Rajendran G, Shanmuganandam K, Bendre A, Muzumdar D, Mujumdar D, Goel A, et al. Epigenetic regulation of DNA methyltransferases: DNMT1 and DNMT3B in gliomas. J Neurooncol. 2011 Sep;104(2):483–94.

55. Li A, Omura N, Hong S-M, Goggins M. Pancreatic cancer DNMT1 expression and sensitivity to DNMT1 inhibitors. Cancer Biol Ther [Internet]. 2010 Feb 25 [cited 2013 Jan 8];9(4). Available from: http://www.ncbi.nlm.nih.gov/pmc/articles/PMC2920347/

56. Girault I, Tozlu S, Lidereau R, Bièche I. Expression Analysis of DNA Methyltransferases 1, 3A, and 3B in Sporadic Breast Carcinomas. Clin Cancer Res. 2003 Oct 1;9(12):4415–22.

57. Kanai Y, Ushijima S, Kondo Y, Nakanishi Y, Hirohashi S. DNA methyltransferase expression and DNA methylation of CPG islands and peri-centromeric satellite regions in human colorectal and stomach cancers. International Journal of Cancer. 2001;91(2):205–12.

58. Festuccia C. Increased levels of DNA methyltransferases are associated with the tumorigenic capacity of prostate cancer cells. Oncology Reports [Internet]. 2012 Dec 18 [cited 2013 Jan 1]; Available from: http://www.spandidos-publications.com/10.3892/or.2012.2192

59. Amara K, Ziadi S, Hachana M, Soltani N, Korbi S, Trimeche M. DNA methyltransferase DNMT3b protein overexpression as a prognostic factor in patients with diffuse large B-cell lymphomas. Cancer Science. 2010;101(7):1722–30.

60. Minami K, Chano T, Kawakami T, Ushida H, Kushima R, Okabe H, et al. DNMT3L Is a Novel Marker and Is Essential for the Growth of Human Embryonal Carcinoma. Clin Cancer Res. 2010 May 15;16(10):2751–9.

61. Gokul G, Gautami B, Malathi S, Sowjanya AP, Poli UR, Jain M, et al. DNA Methylation Profile at the DNMT3L Promoter. Epigenetics. 2007;2(2):80–5.

62. Cho H-S, Kelly JD, Hayami S, Toyokawa G, Takawa M, Yoshimatsu M, et al. Enhanced expression of EHMT2 is involved in the proliferation of cancer cells through negative regulation of SIAH1. Neoplasia. 2011 Aug;13(8):676–84.

63. Candelaria M, de la Cruz-Hernandez E, Taja-Chayeb L, Perez-Cardenas E, Trejo-Becerril C, Gonzalez-Fierro A, et al. DNA Methylation-Independent Reversion of Gemcitabine Resistance by Hydralazine in Cervical Cancer Cells. PLoS ONE. 2012 Mar 12;7(3):e29181.

64. Chen M-W, Hua K-T, Kao H-J, Chi C-C, Wei L-H, Johansson G, et al. H3K9 Histone Methyltransferase G9a Promotes Lung Cancer Invasion and Metastasis by Silencing the Cell Adhesion Molecule Ep-CAM. Cancer Res. 2010 Oct 15;70(20):7830–40.

65. Biankin AV, Waddell N, Kassahn KS, Gingras M-C, Muthuswamy LB, Johns AL, et al. Pancreatic cancer genomes reveal aberrations in axon guidance pathway genes. Nature. 2012 Nov 15;491(7424):399–405.

66. Rice KL, Lin X, Wolniak K, Ebert BL, Berkofsky-Fessler W, Buzzai M, et al. Analysis of genomic aberrations and gene expression profiling identifies novel lesions and pathways in myeloproliferative neoplasms. Blood Cancer J. 2011 Nov;1(11):e40.

67. Ernst T, Chase AJ, Score J, Hidalgo-Curtis CE, Bryant C, Jones AV, et al. Inactivating mutations of the histone methyltransferase gene EZH2 in myeloid disorders. Nature Genetics. 2010;42(8):722–6.

68. Cha J-D, Kim HJ, Cha I-H. Genetic alterations in oral squamous cell carcinoma progression detected by

combining array-based comparative genomic hybridization and multiplex ligation-dependent probe amplification. Oral Surgery, Oral Medicine, Oral Pathology, Oral Radiology, and Endodontology. 2011 May;111(5):594–607.

69. Xie HJ, Noh JH, Kim JK, Jung KH, Eun JW, Bae HJ, et al. HDAC1 Inactivation Induces Mitotic Defect and Caspase-Independent Autophagic Cell Death in Liver Cancer. PLoS One [Internet]. 2012 Apr 4 [cited 2013 Jan 20];7(4). Available from: http://www.ncbi.nlm.nih.gov/pmc/articles/PMC3319574/

70. Eom M, Oh SS, Lkhagvadorj S, Han A, Park KH. HDAC1 Expression in Invasive Ductal Carcinoma of the Breast and Its Value as a Good Prognostic Factor. Korean J Pathol. 2012 Aug;46(4):311–7.

71. Ropero S, Esteller M. The role of histone deacetylases (HDACs) in human cancer. Molecular Oncology. 2007 Jun;1(1):19–25.

72. Wilson AJ, Byun D-S, Popova N, Murray LB, L'Italien K, Sowa Y, et al. Histone Deacetylase 3 (HDAC3) and Other Class I HDACs Regulate Colon Cell Maturation and p21 Expression and Are Deregulated in Human Colon Cancer. J Biol Chem. 2006 May 12;281(19):13548–58.

73. Weichert W, Röske A, Gekeler V, Beckers T, Stephan C, Jung K, et al. Histone deacetylases 1, 2 and 3 are highly expressed in prostate cancer and HDAC2 expression is associated with shorter PSA relapse time after radical prostatectomy. Br J Cancer. 2008 Feb 12;98(3):604–10.

74. Quint K, Agaimy A, Di Fazio P, Montalbano R, Steindorf C, Jung R, et al. Clinical significance of histone deacetylases 1, 2, 3, and 7: HDAC2 is an independent predictor of survival in HCC. Virchows Arch. 2011 Aug;459(2):129–39.

75. Jung KH, Noh JH, Kim JK, Eun JW, Bae HJ, Xie HJ, et al. HDAC2 overexpression confers oncogenic potential to human lung cancer cells by deregulating expression of apoptosis and cell cycle proteins. Journal of Cellular Biochemistry. 2012;113(6):2167–77.

76. Huang BH, Laban M, Leung CH-W, Lee L, Lee CK, Salto-Tellez M, et al. Inhibition of histone deacetylase 2 increases apoptosis and p21Cip1/WAF1 expression, independent of histone deacetylase 1. Cell Death & Differentiation. 2005;12(4):395–404.

77. Weichert W, Denkert C, Noske A, Darb-Esfahani S, Dietel M, Kalloger SE, et al. Expression of Class I Histone Deacetylases Indicates Poor Prognosis in Endometrioid Subtypes of Ovarian and Endometrial Carcinomas. Neoplasia. 2008 Sep;10(9):1021–7.

78. Zhang X, Zhao X, Fiskus W, Lin J, Lwin T, Rao R, et al. Coordinated Silencing of MYC-Mediated miR-29 by HDAC3 and EZH2 as a Therapeutic Target of Histone Modification in Aggressive B-Cell Lymphomas. Cancer Cell. 2012 Oct 16;22(4):506–23.

79. Van Damme M, Crompot E, Meuleman N, Mineur P, Bron D, Lagneaux L, et al. HDAC isoenzyme expression is deregulated in chronic lymphocytic leukemia B-cells and has a complex prognostic significance. Epigenetics [Internet]. 2012 Dec 1 [cited 2013 Jan 1];7(12). Available from: http://www.landesbioscience.com/journals/epigenetics/article/22674/?nocache=624698167

80. Stark M, Hayward N. Genome-Wide Loss of Heterozygosity and Copy Number Analysis in Melanoma Using High-Density Single-Nucleotide Polymorphism Arrays. Cancer Res. 2007 Mar 15;67(6):2632–42.

81. Sjöblom T, Jones S, Wood LD, Parsons DW, Lin J, Barber TD, et al. The Consensus Coding Sequences of Human Breast and Colorectal Cancers. Science. 2006 Oct 13;314(5797):268–74.

82. Moreno DA, Scrideli CA, Cortez MAA, De Paula Queiroz R, Valera ET, Da Silva Silveira V, et al. research paper: Differential expression of HDAC3, HDAC7 and HDAC9 is associated with prognosis and survival in childhood acute lymphoblastic leukaemia. British Journal of Haematology. 2010;150(6):665–73.

83. Stronach EA, Alfraidi A, Rama N, Datler C, Studd J, Agarwal R, et al. HDAC4-regulated STAT1 activation mediates platinum resistance in ovarian cancer. Cancer Res. 2011 Jul 1;71(13):4412–22.

84. Milde T, Oehme I, Korshunov A, Kopp-Schneider A, Remke M, Northcott P, et al. HDAC5 and HDAC9 in Medulloblastoma: Novel Markers for Risk Stratification and Role in Tumor Cell Growth. Clin Cancer Res. 2010 Jun 15;16(12):3240–52.

85. Maesawa C. Overexpression of histone deacetylase 6 contributes to accelerated migration and invasion activity of hepatocellular carcinoma cells. Oncology Reports [Internet]. 2012 Jul 4 [cited 2013 Jan 1]; Available from: http://www.spandidos-publications.com/or/28/3/867

86. Wang L, Xiang S, Williams KA, Dong H, Bai W, Nicosia SV, et al. Depletion of HDAC6 Enhances Cisplatin-Induced DNA Damage and Apoptosis in Non-Small Cell Lung Cancer Cells. PLoS One [Internet]. 2012 Sep 5 [cited 2013 Jan 1];7(9). Available from: http://www.ncbi.nlm.nih.gov/pmc/articles/PMC3434198/

87. Zhang Z, Yamashita H, Toyama T, Sugiura H, Omoto Y, Ando Y, et al. HDAC6 Expression Is Correlated with Better Survival in Breast Cancer. Clin Cancer Res. 2004 Oct 15;10(20):6962–8.

88. Ouaïssi M, Sielezneff I, Silvestre R, Sastre B, Bernard J-P, Lafontaine JS, et al. High histone deacetylase 7 (HDAC7) expression is significantly associated with adenocarcinomas of the pancreas. Ann Surg Oncol. 2008 Aug;15(8):2318–28.

89. Oehme I, Deubzer HE, Wegener D, Pickert D, Linke J-P, Hero B, et al. Histone deacetylase 8 in neuroblastoma tumorigenesis. Clin Cancer Res. 2009 Jan 1;15(1):91–9.

90. Fonseca AL, Kugelberg J, Starker LF, Scholl U, Choi M, Hellman P, et al. Comprehensive DNA methylation analysis of benign and malignant adrenocortical tumors. Genes Chromosomes Cancer. 2012 Oct;51(10):949–60.

91. Wang JC, Kafeel MI, Avezbakiyev B, Chen C, Sun Y, Rathnasabapathy C, et al. Histone deacetylase in chronic lymphocytic leukemia. Oncology. 2011;81(5-6):325–9.

92. Osada H, Tatematsu Y, Saito H, Yatabe Y, Mitsudomi T, Takahashi T. Reduced expression of class II histone deacetylase genes is associated with poor prognosis in lung cancer patients. Int J Cancer. 2004 Oct 20;112(1):26–32.

93. Rinner B, Gallè B, Trajanoski S, Fischer C, Hatz M, Maierhofer T, et al. Molecular evidence for the bi-clonal origin of neuroendocrine tumor derived metastases. BMC Genomics. 2012 Nov 5;13:594.

94. Rebouissou S, Rosty C, Lecuru F, Boisselier S, Bui H, Le Frere-Belfa M-A, et al. Mutation of TCF1 encoding hepatocyte nuclear factor 1alpha in gynecological cancer. Oncogene. 2004 Sep 30;23(45):7588–92.

95. Laurent-Puig P, Plomteux O, Bluteau O, Zinzindohoué F, Jeannot E, Dahan K, et al. Frequent mutations of hepatocyte nuclear factor 1 in colorectal cancer with microsatellite instability. Gastroenterology. 2003 May;124(5):1311–4.

96. Jeannot E, Poussin K, Chiche L, Bacq Y, Sturm N, Scoazec J-Y, et al. Association of CYP1B1 Germ Line Mutations with Hepatocyte Nuclear Factor 1α–Mutated Hepatocellular Adenoma. Cancer Res. 2007 Mar 15;67(6):2611–6.

97. Wang W, Hayashi Y, Ninomiya T, Ohta K, Nakabayashi H, Tamaoki T, et al. Expression of HNF-1 alpha and HNF-1 beta in various histological differentiations of hepatocellular carcinoma. J Pathol. 1998 Mar;184(3):272–8.

98. Mullighan CG, Su X, Zhang J, Radtke I, Phillips LAA, Miller CB, et al. Deletion of IKZF1 and Prognosis in Acute Lymphoblastic Leukemia. N Engl J Med. 2009 Jan 29;360(5):470–80.

99. Gunduz M, Nagatsuka H, Demircan K, Gunduz E, Cengiz B, Ouchida M, et al. Frequent deletion and down-regulation of ING4, a candidate tumor suppressor gene at 12p13, in head and neck squamous cell carcinomas. Gene. 2005 Aug 15;356:109–17.

100. Li J, Martinka M, Li G. Role of ING4 in human melanoma cell migration, invasion and patient survival. Carcinogenesis. 2008 Jul;29(7):1373–9.

101. Li M, Jin Y, Sun W, Yu Y, Bai J, Tong D, et al. Reduced expression and novel splice variants of ING4 in human gastric adenocarcinoma. J Pathol. 2009 Sep;219(1):87–95.

102. Wang Q, Li M, Zhang L, Jin Y, Tong D, Yu Y, et al. Down-regulation of ING4 is associated with initiation and progression of lung cancer. Histopathology. 2010 Aug;57(2):271–81.

103. You Q, Wang X-S, Fu S-B, Jin X-M. Downregulated expression of inhibitor of growth 4 (ING4) in advanced colorectal cancers: a non-randomized experimental study. Pathol Oncol Res. 2011 Sep;17(3):473–7.

104. Tapia C, Zlobec I, Schneider S, Kilic E, Güth U, Bubendorf L, et al. Deletion of the inhibitor of growth 4 (ING4) tumor suppressor gene is prevalent in human epidermal growth factor 2 (HER2)-positive breast cancer. Hum Pathol. 2011 Jul;42(7):983–90.

105. Manceau G, Letouzé E, Guichard C, Didelot A, Cazes A, Corté H, et al. Recurrent inactivating mutations of ARID2 in non-small cell lung carcinoma. Int J Cancer. 2012 Oct 10;

106. Tzatsos A, Paskaleva P, Ferrari F, Deshpande V, Stoykova S, Contino G, et al. KDM2B promotes pancreatic cancer via Polycomb-dependent and -independent transcriptional programs. J Clin Invest. 2013 Feb 1;123(2):727–39.

107. Lee YF, Miller LD, Chan XB, Black MA, Pang B, Ong CW, et al. JMJD6 is a driver of cellular proliferation and motility and a marker of poor prognosis in breast cancer. Breast Cancer Res. 2012;14(3):R85.

108. Sakuraba K, Yokomizo K, Shirahata A, Goto T, Saito M, Ishibashi K, et al. TIP60 as a potential marker for the malignancy of gastric cancer. Anticancer Res. 2011 Jan;31(1):77–9.

109. Chen G, Cheng Y, Tang Y, Martinka M, Li G. Role of Tip60 in human melanoma cell migration, metastasis, and patient survival. J Invest Dermatol. 2012 Nov;132(11):2632–41.

110. Sakuraba K, Yasuda T, Sakata M, Kitamura Y-H, Shirahata A, Goto T, et al. Down-regulation of Tip60 Gene as a Potential Marker for the Malignancy of Colorectal Cancer. Anticancer Res. 2009 Oct 1;29(10):3953–5.

111. Wood LD, Parsons DW, Jones S, Lin J, Sjöblom T, Leary RJ, et al. The Genomic Landscapes of Human Breast and Colorectal Cancers. Science. 2007 Nov 16;318(5853):1108–13.

112. Moore SDP, Herrick SR, Ince TA, Kleinman MS, Cin PD, Morton CC, et al. Uterine Leiomyomata with t(10;17) Disrupt the Histone Acetyltransferase MORF. Cancer Res. 2004 Aug 15;64(16):5570–7.

113. Iizuka M, Takahashi Y, Mizzen CA, Cook RG, Fujita M, Allis CD, et al. Histone acetyltransferase Hbo1: catalytic activity, cellular abundance, and links to primary cancers. Gene. 2009 May 1;436(1-2):108–14.

114. Pfister S, Rea S, Taipale M, Mendrzyk F, Straub B, Ittrich C, et al. The histone acetyltransferase hMOF is frequently downregulated in primary breast carcinoma and medulloblastoma and constitutes a biomarker for clinical outcome in medulloblastoma. Int J Cancer. 2008 Mar 15;122(6):1207–13.

115. Lv T, Yuan D, Miao X, Lv Y, Zhan P, Shen X, et al. Over-Expression of LSD1 Promotes Proliferation, Migration and Invasion in Non-Small Cell Lung Cancer. PLoS One [Internet]. 2012 Apr 6 [cited 2013 Jan 20];7(4). Available from: http://www.ncbi.nlm.nih.gov/pmc/articles/PMC3320866/

116. Schildhaus H-U, Riegel R, Hartmann W, Steiner S, Wardelmann E, Merkelbach-Bruse S, et al. Lysine-specific demethylase 1 is highly expressed in solitary fibrous tumors, synovial sarcomas, rhabdomyosarcomas, desmoplastic small round cell tumors, and malignant peripheral nerve sheath tumors. Hum Pathol. 2011 Nov;42(11):1667–75.

117. Hayami S, Kelly JD, Cho H-S, Yoshimatsu M, Unoki M, Tsunoda T, et al. Overexpression of LSD1 contributes to human carcinogenesis through chromatin regulation in various cancers. Int J Cancer. 2011 Feb 1;128(3):574–86.

118. Kahl P, Gullotti L, Heukamp LC, Wolf S, Friedrichs N, Vorreuther R, et al. Androgen Receptor Coactivators Lysine-Specific Histone Demethylase 1 and Four and a Half LIM Domain Protein 2 Predict Risk of Prostate Cancer Recurrence. Cancer Res. 2006 Dec 1;66(23):11341–7.

119. Wang Y, Zhang H, Chen Y, Sun Y, Yang F, Yu W, et al. LSD1 is a subunit of the NuRD complex and targets the metastasis programs in breast cancer. Cell. 2009 Aug 21;138(4):660–72.

120. Frescas D, Guardavaccaro D, Kato H, Poleshko A, Katz RA, Pagano M. KDM2A represses transcription of centromeric satellite repeats and maintains the heterochromatic state. Cell Cycle. 2008 Nov 15;7(22):3539–47.

121. He J, Nguyen AT, Zhang Y. KDM2b/JHDM1b, an H3K36me2-specific demethylase, is required for initiation and maintenance of acute myeloid leukemia. Blood. 2011 Apr 7;117(14):3869–80.

122. Björkman M, Östling P, Härmä V, Virtanen J, Mpindi J-P, Rantala J, et al. Systematic knockdown of epigenetic enzymes identifies a novel histone demethylase PHF8 overexpressed in prostate cancer with an impact on cell proliferation, migration and invasion. Oncogene. 2012 Jul 19;31(29):3444–56.

123. Guo X, Shi M, Sun L, Wang Y, Gui Y, Cai Z, et al. The expression of histone demethylase JMJD1A in renal cell carcinoma. Neoplasma. 2011;58(2):153–7.

124. Kim J-Y, Kim K-B, Eom GH, Choe N, Kee HJ, Son H-J, et al. KDM3B Is the H3K9 Demethylase Involved in Transcriptional Activation of lmo2 in Leukemia. Mol Cell Biol. 2012 Jul;32(14):2917–33.

125. Berry WL, Shin S, Lightfoot SA, Janknecht R. Oncogenic features of the JMJD2A histone demethylase in breast cancer. Int J Oncol. 2012 Nov;41(5):1701–6.

126. Kauffman EC, Robinson BD, Downes M, Powell LG, Lee MM, Scherr DS, et al. Role of androgen receptor and associated lysine-demethylase coregulators, LSD1 and JMJD2A, in localized and advanced human bladder cancer. Mol Carcinog. 2011 Dec;50(12):931–44.

127. Li W, Zhao L, Zang W, Liu Z, Chen L, Liu T, et al. Histone demethylase JMJD2B is required for tumor cell proliferation and survival and is overexpressed in gastric cancer. Biochem Biophys Res Commun. 2011 Dec 16;416(3-4):372–8.

128. Liu G, Bollig-Fischer A, Kreike B, van de Vijver MJ, Abrams J, Ethier SP, et al. Genomic amplification and oncogenic properties of the GASC1 histone demethylase gene in breast cancer. Oncogene. 2009 Dec 17;28(50):4491–500.

129. Roesch A, Becker B, Meyer S, Wild P, Hafner C, Landthaler M, et al. Retinoblastoma-binding protein 2-homolog 1: a retinoblastoma-binding protein downregulated in malignant melanomas. Modern Pathology. 2005;18(9):1249–57.

130. Paolicchi E, Crea F, Farrar WL, Green JE, Danesi R. Histone lysine demethylases in breast cancer. Critical Reviews in Oncology/Hematology [Internet]. 2012 Dec [cited 2013 Jan 2]; Available from: http://www.croh-online.com/article/S1040-8428(12)00235-1/fulltext

131. Radberger P, Radberger A, Bykov VJN, Seregard S, Economou MA. JARID1B protein expression and prognostic implications in uveal melanoma. Invest Ophthalmol Vis Sci. 2012 Jul;53(8):4442–9.

132. Anderton JA, Bose S, Vockerodt M, Vrzalikova K, Wei W, Kuo M, et al. The H3K27me3 demethylase, KDM6B, is induced by Epstein-Barr virus and over-expressed in Hodgkin's Lymphoma. Oncogene. 2011 Apr 28;30(17):2037–43.

133. Willis ND, Cox TR, Rahman-Casañs SF, Smits K, Przyborski SA, van den Brandt P, et al. Lamin A/C Is a Risk Biomarker in Colorectal Cancer. PLoS ONE [Internet]. 2008 Aug 20 [cited 2013 Jan 20];3(8).

Available from: http://www.ncbi.nlm.nih.gov/pmc/articles/PMC2496895/

134. Agrelo R, Setien F, Espada J, Artiga MJ, Rodriguez M, Pérez-Rosado A, et al. Inactivation of the Lamin A/C Gene by CpG Island Promoter Hypermethylation in Hematologic Malignancies, and Its Association With Poor Survival in Nodal Diffuse Large B-Cell Lymphoma. JCO. 2005 Jun 10;23(17):3940–7.

135. Stadelmann B, Khandjian E, Hirt A, Lüthy A, Weil R, Wagner HP. Repression of nuclear lamin A and C gene expression in human acute lymphoblastic leukemia and non-Hodgkin's lymphoma cells. Leuk Res. 1990;14(9):815–21.

136. Wong K-F, Luk JM. Discovery of lamin B1 and vimentin as circulating biomarkers for early hepatocellular carcinoma. Methods Mol Biol. 2012;909:295–310.

137. Marshall KW, Mohr S, Khettabi FE, Nossova N, Chao S, Bao W, et al. A blood-based biomarker panel for stratifying current risk for colorectal cancer. International Journal of Cancer. 2010;126(5):1177–86.

138. Somatic frameshift mutations in the MBD4 gene of sporadic colon cancers with mismatch repair deficiency. , Published online: 04 January 2000; | doi:101038/sj.onc1203229 [Internet]. 2000 Jan 4 [cited 2013 Jan 20];18(56). Available from: http://www.nature.com/onc/journal/v18/n56/full/1203229a.html

139. Riccio A, Aaltonen LA, Godwin AK, Loukola A, Percesepe A, Salovaara R, et al. The DNA repair gene MBD4 (MED1) is mutated in human carcinomas with microsatellite instability. Nature Genetics. 1999;23(3):266–8.

140. Müller HM, Fiegl H, Goebel G, Hubalek MM, Widschwendter A, Müller-Holzner E, et al. MeCP2 and MBD2 expression in human neoplastic and non-neoplastic breast tissue and its association with oestrogen receptor status. Br J Cancer. 2003 Nov 17;89(10):1934–9.

141. Debelenko LV, Brambilla E, Agarwal SK, Swalwell JI, Kester MB, Lubensky IA, et al. Identification of MEN1 Gene Mutations in Sporadic Carcinoid Tumors of the Lung. Hum Mol Genet. 1997 Dec 1;6(13):2285–90.

142. Thiel AT, Huang J, Lei M, Hua X. Menin as a hub controlling mixed lineage leukemia. BioEssays. 2012;34(9):771–80.

143. Damm F, Oberacker T, Thol F, Surdziel E, Wagner K, Chaturvedi A, et al. Prognostic importance of histone methyltransferase MLL5 expression in acute myeloid leukemia. J Clin Oncol. 2011 Feb 20;29(6):682–9.

144. Li D-Q, Pakala SB, Nair SS, Eswaran J, Kumar R. Metastasis Associated Protein 1/Nucleosome Remodeling and Histone Deacetylase Complex in Cancer. Cancer Res. 2012 Jan 15;72(2):387–94.

145. Liu S-L, Han Y, Zhang Y, Xie C-Y, Wang E-H, Miao Y, et al. Expression of metastasis-associated protein 2 (MTA2) might predict proliferation in non-small cell lung cancer. Target Oncol. 2012 Jun;7(2):135–43.

146. Lee H, Ryu SH, Hong SS, Seo DD, Min HJ, Jang MK, et al. Overexpression of metastasis-associated protein 2 is associated with hepatocellular carcinoma size and differentiation. J Gastroenterol Hepatol. 2009 Aug;24(8):1445–50.

147. Ji Y, Zhang P, Lu Y, Ma D. Expression of MTA2 gene in ovarian epithelial cancer and its clinical implication. J Huazhong Univ Sci Technol Med Sci. 2006;26(3):359–62.

148. Sundram U, Kim Y, Mraz-Gernhard S, Hoppe R, Natkunam Y, Kohler S. Expression of the bcl-6 and MUM1/IRF4 proteins correlate with overall and disease-specific survival in patients with primary cutaneous large B-cell lymphoma: a tissue microarray study. J Cutan Pathol. 2005 Mar;32(3):227–34.

149. Ito M, Iida S, Inagaki H, Tsuboi K, Komatsu H, Yamaguchi M, et al. MUM1/IRF4 expression is an unfavorable prognostic factor in B-cell chronic lymphocytic leukemia (CLL)/small lymphocytic lymphoma (SLL). Jpn J Cancer Res. 2002 Jun;93(6):685–94.

150. Tsuboi K, Iida S, Inagaki H, Kato M, Hayami Y, Hanamura I, et al. MUM1/IRF4 expression as a frequent event in mature lymphoid malignancies. Leukemia. 2000 Mar;14(3):449–56.

151. Xu Y, Chen Q, Li W, Su X, Chen T, Liu Y, et al. Overexpression of transcriptional coactivator AIB1 promotes hepatocellular carcinoma progression by enhancing cell proliferation and invasiveness. Oncogene. 2010;29(23):3386–97.

152. Luo J-H, Xie D, Liu M-Z, Chen W, Liu Y-D, Wu G-Q, et al. Protein expression and amplification of AIB1 in human urothelial carcinoma of the bladder and overexpression of AIB1 is a new independent prognostic marker of patient survival. International Journal of Cancer. 2008;122(11):2554–61.

153. He L-R, Zhao H-Y, Li B-K, Zhang L-J, Liu M-Z, Kung H-F, et al. Overexpression of AIB1 negatively affects survival of surgically resected non-small-cell lung cancer patients. Ann Oncol. 2010 Aug 1;21(8):1675–81.

154. Zhou H-J, Yan J, Luo W, Ayala G, Lin S-H, Erdem H, et al. SRC-3 Is Required for Prostate Cancer Cell Proliferation and Survival. Cancer Res. 2005 Sep 1;65(17):7976–83.

155. Gojis O, Rudraraju B, Gudi M, Hogben K, Sousha S, Coombes CR, et al. The role of SRC-3 in human breast cancer. Nature Reviews Clinical Oncology. 2009 Dec 22;7(2):83–9.

156. Esteyries S, Perot C, Adelaide J, Imbert M, Lagarde A, Pautas C, et al. NCOA3, a new fusion partner for MOZ/MYST3 in M5 acute myeloid leukemia. Leukemia. 2008;22(3):663–5.

157. Zakrzewska M, Zakrzewski K, Grešner SM, Piaskowski S, Zalewska-Szewczyk B, Liberski PP. Polycomb genes expression as a predictor of poor clinical outcome in children with medulloblastoma. Childs Nerv Syst. 2011 Jan;27(1):79–86.

158. Guo B-H, Zhang X, Zhang H-Z, Lin H-L, Feng Y, Shao J-Y, et al. Low expression of Mel-18 predicts poor prognosis in patients with breast cancer. Ann Oncol. 2010 Dec 1;21(12):2361–9.

159. Wang W, Yuasa T, Tsuchiya N, Ma Z, Maita S, Narita S, et al. The novel tumor-suppressor Mel-18 in prostate cancer: its functional polymorphism, expression and clinical significance. Int J Cancer. 2009 Dec 15;125(12):2836–43.

160. Piao Z, Fang W, Malkhosyan S, Kim H, Horii A, Perucho M, et al. Frequent Frameshift Mutations of RIZ in Sporadic Gastrointestinal and Endometrial Carcinomas with Microsatellite Instability. Cancer Res. 2000 Sep 1;60(17):4701–4.

161. Chadwick RB, Jiang G-L, Bennington GA, Yuan B, Johnson CK, Stevens MW, et al. Candidate tumor suppressor RIZ is frequently involved in colorectal carcinogenesis. Proc Natl Acad Sci U S A. 2000 Mar 14;97(6):2662–7.

162. Poetsch M, Dittberner T, Woenckhaus C. Frameshift mutations of RIZ, but no point mutations in RIZ1 exons in malignant melanomas with deletions in 1p36. Oncogene. 2002 May 2;21(19):3038–42.

163. Sasaki O, Meguro K, Tohmiya Y, Funato T, Shibahara S, Sasaki T. Altered expression of retinoblastoma protein-interacting zinc finger gene, RIZ, in human leukaemia. Br J Haematol. 2002 Dec;119(4):940–8.

164. Dong S-W, Cui Y-T, Zhong R-R, Liang D-C, Liu Y-M, Wang Y-G, et al. Decreased expression of retinoblastoma protein-interacting zinc-finger gene 1 in human esophageal squamous cell cancer by DNA methylation. Clin Lab. 2012;58(1-2):41–51.

165. Geli J, Kiss N, Kogner P, Larsson C. Suppression of RIZ in biologically unfavourable neuroblastomas. Int J Oncol. 2010 Nov;37(5):1323–30.

166. Zhang C, Li H, Wang Y, Liu W, Zhang Q, Zhang T, et al. Epigenetic inactivation of the tumor suppressor gene RIZ1 in hepatocellular carcinoma involves both DNA methylation and histone modifications. J Hepatol. 2010 Nov;53(5):889–95.

167. Akahira J-I, Suzuki F, Suzuki T, Miura I, Kamogawa N, Miki Y, et al. Decreased expression of RIZ1 and its clinicopathological significance in epithelial ovarian carcinoma: correlation with epigenetic inactivation by aberrant DNA methylation. Pathol Int. 2007 Nov;57(11):725–33.

168. Lal G, Padmanabha L, Smith BJ, Nicholson RM, Howe JR, O'Dorisio MS, et al. RIZ1 is epigenetically inactivated by promoter hypermethylation in thyroid carcinoma. Cancer. 2006 Dec 15;107(12):2752–9.

169. Carling T, Du Y, Fang W, Correa P, Huang S. Intragenic allelic loss and promoter hypermethylation of the RIZ1 tumor suppressor gene in parathyroid tumors and pheochromocytomas. Surgery. 2003 Dec;134(6):932–939; discussion 939–940.

170. Lohavanichbutr P, Houck J, Fan W, Yueh B, Mendez E, Futran N, et al. Genome-wide gene expression profiles of HPV-positive and HPV-negative oropharyngeal cancer: potential implications for treatment choices. Arch Otolaryngol Head Neck Surg. 2009 Feb;135(2):180–8.

171. Leivo I, Jee KJ, Heikinheimo K, Laine M, Ollila J, Nagy B, et al. Characterization of gene expression in major types of salivary gland carcinomas with epithelial differentiation. Cancer Genet Cytogenet. 2005 Jan 15;156(2):104–13.

172. Bralten LBC, Kloosterhof NK, Gravendeel LAM, Sacchetti A, Duijm EJ, Kros JM, et al. Integrated genomic profiling identifies candidate genes implicated in glioma-genesis and a novel LEO1-SLC12A1 fusion gene. Genes Chromosomes Cancer. 2010 Jun;49(6):509–17.

173. Wang C-L, Wang C-I, Liao P-C, Chen C-D, Liang Y, Chuang W-Y, et al. Discovery of retinoblastoma-associated binding protein 46 as a novel prognostic marker for distant metastasis in nonsmall cell lung cancer by combined analysis of cancer cell secretome and pleural effusion proteome. J Proteome Res. 2009 Oct;8(10):4428–40.

174. Thakur A, Rahman KW, Wu J, Bollig A, Biliran H, Lin X, et al. Aberrant Expression of X-Linked Genes RbAp46, Rsk4, and Cldn2 in Breast Cancer. Mol Cancer Res. 2007 Feb 1;5(2):171–81.

175. Li Q, Dong Q, Wang E. Rsf-1 is overexpressed in non-small cell lung cancers and regulates cyclinD1 expression and ERK activity. Biochem Biophys Res Commun. 2012 Mar 30;420(1):6–10.

176. Liang P-I, Wu L-C, Sheu JJ-C, Wu T-F, Shen K-H, Wang Y-H, et al. Rsf-1/HBXAP overexpression is independent of gene amplification and is associated with poor outcome in patients with urinary bladder urothelial carcinoma. J Clin Pathol. 2012 Sep;65(9):802–7.

177. Liu S, Dong Q, Wang E. Rsf-1 overexpression correlates with poor prognosis and cell proliferation in colon cancer. Tumour Biol. 2012 Oct;33(5):1485–91.

178. Chen T-J, Huang S-C, Huang H-Y, Wei Y-C, Li C-F. Rsf-1/HBXAP overexpression is associated with disease-specific survival of patients with gallbladder carcinoma. APMIS. 2011 Nov;119(11):808–14.

179. Tai H-C, Huang H-Y, Lee S-W, Lin C-Y, Sheu M-J, Chang S-L, et al. Associations of Rsf-1 overexpression with poor therapeutic response and worse survival in patients with nasopharyngeal carcinoma. J Clin Pathol. 2012 Mar;65(3):248–53.

180. Mao T-L, Hsu C-Y, Yen MJ, Gilks B, Sheu JJ-C, Gabrielson E, et al. Expression of Rsf-1, a chromatin-remodeling gene, in ovarian and breast carcinoma. Hum Pathol. 2006 Sep;37(9):1169–75.

181. Shih I-M, Sheu JJ-C, Santillan A, Nakayama K, Yen MJ, Bristow RE, et al. Amplification of a chromatin remodeling gene, Rsf-1/HBXAP, in ovarian carcinoma. Proc Natl Acad Sci U S A. 2005 Sep 27;102(39):14004–9.

182. Jiang Q, Zhang C, Zhu J, Chen Q, Chen Y. The set gene is a potential oncogene in human colorectal adenocarcinoma and oral squamous cell carcinoma. Molecular Medicine Reports [Internet]. 2011 Jul 1 [cited 2013 Feb 14]; Available from: http://www.spandidos-publications.com/mmr/4/5/993

183. Sirma Ekmekci S, G Ekmekci C, Kandilci A, Gulec C, Akbiyik M, Emrence Z, et al. SET oncogene is

upregulated in pediatric acute lymphoblastic leukemia. Tumori. 2012 Apr;98(2):252–6.

184. Yang F, Sun L, Li Q, Han X, Lei L, Zhang H, et al. SET8 promotes epithelial–mesenchymal transition and confers TWIST dual transcriptional activities. EMBO J. 2012 Jan 4;31(1):110–23.

185. Parker H, Rose-Zerilli MJJ, Parker A, Chaplin T, Wade R, Gardiner A, et al. 13q deletion anatomy and disease progression in patients with chronic lymphocytic leukemia. Leukemia. 2011 Mar;25(3):489–97.

186. Yi J, Luo J. SIRT1 and p53, effect on cancer, senescence and beyond. Biochim Biophys Acta. 2010 Aug;1804(8):1684–9.

187. Wang R-H, Sengupta K, Li C, Kim H-S, Cao L, Xiao C, et al. Impaired DNA damage response, genome instability, and tumorigenesis in SIRT1 mutant mice. Cancer Cell. 2008 Oct 7;14(4):312–23.

188. Hiratsuka M, Inoue T, Toda T, Kimura N, Shirayoshi Y, Kamitani H, et al. Proteomics-based identification of differentially expressed genes in human gliomas: down-regulation of SIRT2 gene. Biochem Biophys Res Commun. 2003 Sep 26;309(3):558–66.

189. Zhang Y-Y, Zhou L-M. Sirt3 inhibits hepatocellular carcinoma cell growth through reducing Mdm2-mediated p53 degradation. Biochem Biophys Res Commun. 2012 Jun 22;423(1):26–31.

190. Sebastián C, Zwaans BMM, Silberman DM, Gymrek M, Goren A, Zhong L, et al. The Histone Deacetylase SIRT6 Is a Tumor Suppressor that Controls Cancer Metabolism. Cell. 2012 Dec 7;151(6):1185–99.

191. Ashraf N, Zino S, MacIntyre A, Kingsmore D, Payne AP, George WD, et al. Altered sirtuin expression is associated with node-positive breast cancer. Br J Cancer. 2006 Oct 23;95(8):1056–61.

192. De Nigris F, Cerutti J, Morelli C, Califano D, Chiariotti L, Viglietto G, et al. Isolation of a SIR-like gene, SIR-T8, that is overexpressed in thyroid carcinoma cell lines and tissues. Br J Cancer. 2002 Mar 18;86(6):917–23.

193. Heebøll S, Borre M, Ottosen PD, Andersen CL, Mansilla F, Dyrskjøt L, et al. SMARCC1 expression is upregulated in prostate cancer and positively correlated with tumour recurrence and dedifferentiation. Histol Histopathol. 2008 Sep;23(9):1069–76.

194. Shadeo A, Chari R, Lonergan KM, Pusic A, Miller D, Ehlen T, et al. Up regulation in gene expression of chromatin remodelling factors in cervical intraepithelial neoplasia. BMC Genomics. 2008 Feb 4;9:64.

195. Andersen CL, Christensen LL, Thorsen K, Schepeler T, Sørensen FB, Verspaget HW, et al. Dysregulation of the transcription factors SOX4, CBFB and SMARCC1 correlates with outcome of colorectal cancer. Br J Cancer. 2009 Feb 10;100(3):511–23.

196. Stephens PJ, Tarpey PS, Davies H, Loo PV, Greenman C, Wedge DC, et al. The landscape of cancer genes and mutational processes in breast cancer. Nature. 2012 Jun 21;486(7403):400–4.

197. Takita J, Ishii M, Tsutsumi S, Tanaka Y, Kato K, Toyoda Y, et al. Gene expression profiling and identification of novel prognostic marker genes in neuroblastoma. Genes Chromosomes Cancer. 2004 Jun;40(2):120–32.

198. Kagami S, Kurita T, Kawagoe T, Toki N, Matsuura Y, Hachisuga T, et al. Prognostic significance of BAF57 expression in patients with endometrial carcinoma. Histol Histopathol. 2012 May;27(5):593–9.

199. Komatsu S, Imoto I, Tsuda H, Kozaki K, Muramatsu T, Shimada Y, et al. Overexpression of SMYD2 relates to tumor cell proliferation and malignant outcome of esophageal squamous cell carcinoma. Carcinogenesis. 2009 Jul 1;30(7):1139–46.

200. Xi Y, Formentini A, Nakajima G, Kornmann M, Ju J. Validation of biomarkers associated with 5-fluorouracil and thymidylate synthase in colorectal cancer. Oncol Rep. 2008 Jan;19(1):257–62.

201. Li H, Cai Q, Wu H, Vathipadiekal V, Dobbin ZC, Li T, et al. SUZ12 promotes human epithelial ovarian cancer by suppressing apoptosis via silencing HRK. Mol Cancer Res. 2012 Nov;10(11):1462–72.

202. Martín-Pérez D, Sánchez E, Maestre L, Suela J, Vargiu P, Di Lisio L, et al. Deregulated Expression of the Polycomb-Group Protein SUZ12 Target Genes Characterizes Mantle Cell Lymphoma. Am J Pathol. 2010 Aug;177(2):930–42.

203. Liu Y, Sun W, Zhang K, Zheng H, Ma Y, Lin D, et al. Identification of genes differentially expressed in human primary lung squamous cell carcinoma. Lung Cancer. 2007 Jun;56(3):307–17.

204. Parker H, An Q, Barber K, Case M, Davies T, Konn Z, et al. The complex genomic profile of ETV6-RUNX1 positive acute lymphoblastic leukemia highlights a recurrent deletion of TBL1XR1. Genes Chromosomes Cancer. 2008 Dec;47(12):1118–25.

205. Braggio E, McPhail ER, Macon W, Lopes MB, Schiff D, Law M, et al. Primary Central Nervous System Lymphomas: A Validation Study of Array-Based Comparative Genomic Hybridization in Formalin-Fixed Paraffin-Embedded Tumor Specimens. Clin Cancer Res. 2011 Jul 1;17(13):4245–53.

206. Kalender Atak Z, De Keersmaecker K, Gianfelici V, Geerdens E, Vandepoel R, Pauwels D, et al. High Accuracy Mutation Detection in Leukemia on a Selected Panel of Cancer Genes. PLoS One [Internet]. 2012 Jun 4 [cited 2013 Jan 1];7(6). Available from: http://www.ncbi.nlm.nih.gov/pmc/articles/PMC3366948/

207. Hsu C-H, Peng K-L, Kang M-L, Chen Y-R, Yang Y-C, Tsai C-H, et al. TET1 Suppresses Cancer Invasion by Activating the Tissue Inhibitors of Metalloproteinases. Cell Reports. 2012 Sep 27;2(3):568–79.

208. Kijanka G, Hector S, Kay EW, Murray F, Cummins R, Murphy D, et al. Human IgG antibody profiles differentiate between symptomatic patients with and without colorectal cancer. Gut. 2010 Jan;59(1):69–78.

209. Yokoe T, Toiyama Y, Okugawa Y, Tanaka K, Ohi M, Inoue Y, et al. KAP1 is associated with peritoneal carcinomatosis in gastric cancer. Ann Surg Oncol. 2010 Mar;17(3):821–8.

210. Chen L, Chen D-T, Kurtyka C, Rawal B, Fulp WJ, Haura EB, et al. Tripartite Motif Containing 28 (Trim28) Can Regulate Cell Proliferation by Bridging HDAC1/E2F Interactions. J Biol Chem. 2012 Nov 23;287(48):40106–18.

211. Atchison M, Basu A, Zaprazna K, Papasani M. Mechanisms of Yin Yang 1 in Oncogenesis: The Importance of Indirect Effects. Crit Rev Oncog. 2011;16(3-4):143–61.

212. Lister R, Pelizzola M, Dowen RH, Hawkins RD, Hon G, Tonti-Filippini J, et al. Human DNA methylomes at base resolution show widespread epigenomic differences. Nature [Internet]. 2009 Oct 14 [cited 2009 Nov 4];advance online publication. Available from: http://dx.doi.org/10.1038/nature08514

213. Viré E, Brenner C, Deplus R, Blanchon L, Fraga M, Didelot C, et al. The Polycomb group protein EZH2 directly controls DNA methylation. Nature. 2005 Dec 14;439(7078):871–4.

214. The ENCODE Project Consortium. Identification and analysis of functional elements in 1% of the human genome by the ENCODE pilot project. Nature. 2007 Jun 14;447(7146):799–816.

215. Barski A, Cuddapah S, Cui K, Roh T-Y, Schones DE, Wang Z, et al. High-Resolution Profiling of Histone Methylations in the Human Genome. Cell. 2007 May 18;Vol 129:823–37.

216. Hansen RS, Thomas S, Sandstrom R, Canfield TK, Thurman RE, Weaver M, et al. Sequencing newly replicated DNA reveals widespread plasticity in human replication timing. Proceedings of the National Academy of Sciences. 2010 Jan 5;107(1):139 –144.
