# Supplementary material for: The mutational landscape of chromatin regulatory factors across 4,623 tumor samples
Source: Genome Biol. 2013 Sep 24;14(9):r106. doi: 10.1186/gb-2013-14-9-r106 (PMC4054018; doi:10.1186/gb-2013-14-9-r106)
Supplement: Additional file 2 — Supplementary Figure S1 and S2. [file gb-2013-14-9-r106-S2.pdf]

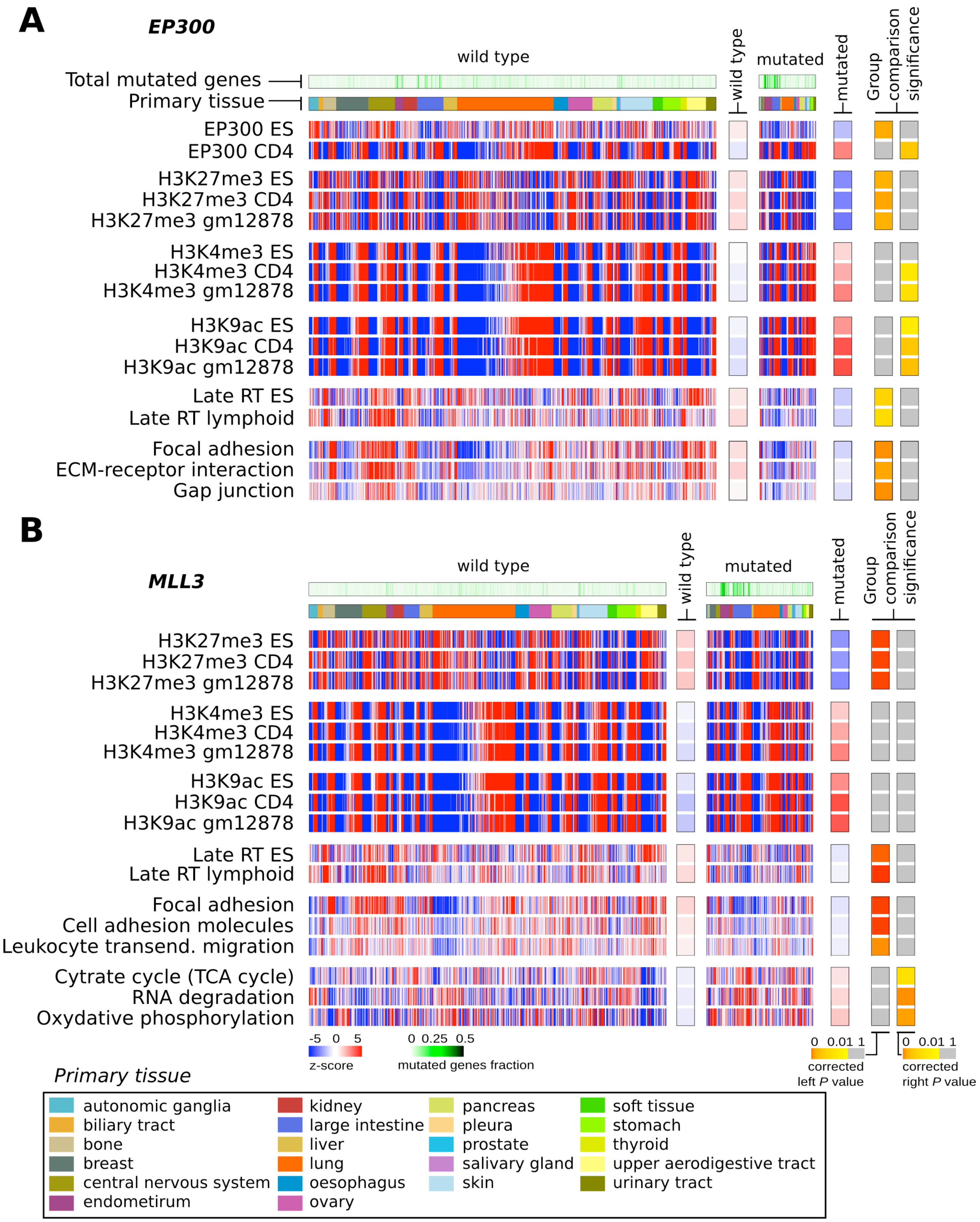

Figure S1. Effect of PAMs in *EP300* and *MLL3* on regulatory modules transcription across cancer cell lines. Cancer cell lines originated from solid tissues (see "primary tissue" colour legend) are enriched (SLEA) for regulatory modules (see Table S1 in Additional file 3) and selected pathways from KEGG. Left and right SLEA panels correspond to cells wild type or with a protein affecting mutation, respectively. The difference between the two enrichment groups, assessed with a Wilcoxon-Mann-Whitney group comparison test, is indicated on the right. *A*. *EP300* mutation status. *B*. *MLL3* mutation status.

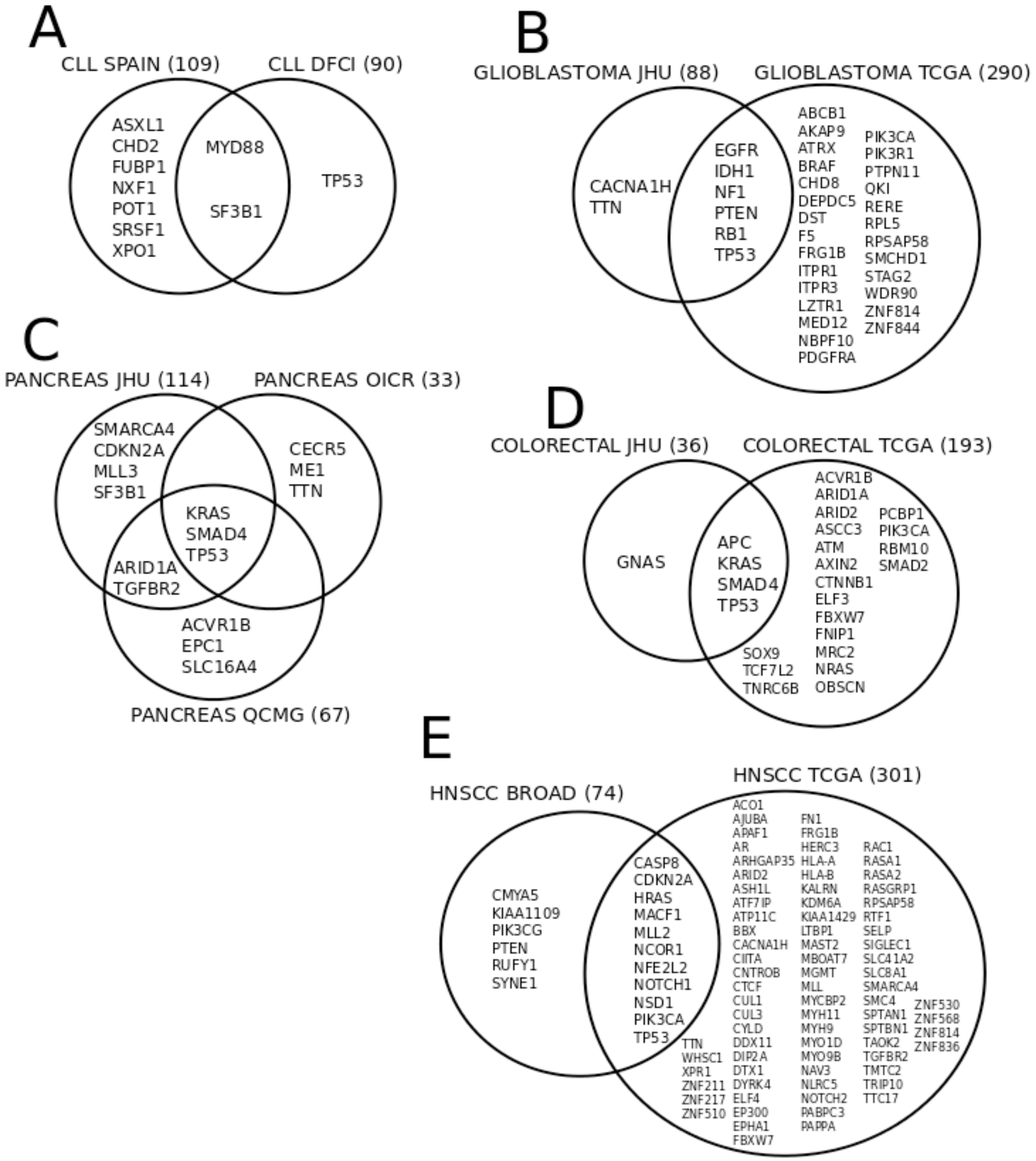

Figure S2. Overlap of drivers from different datasets of the same tumor type. We examined the overlap of the lists of predicted driver genes obtained from different datasets of somatic mutations of the same tumor type. Specifically, we looked at five cases where we had in IntOGen more than one dataset of somatic mutations comprising samples from more the same disease. In the figure, below, these are presented in the following order: A, two datasets of chronic lymphocytic leukemia; B, two datasets of glioblastoma multiforme; C, three datasets of pancreatic cancer; D, two datasets of colorectal adenocarcinoma; E, two datasets of head and neck squamous cell carcinoma. The overlaps are represented through Venn diagrams, containing the symbols of genes shared by the datasets or unique to each of them. The name of each dataset is accompanied by the number of samples it contains in parentheses.
